# Glucagon Reduces Neutrophil Migration and Increases Susceptibility to Sepsis in Diabetic Mice

**DOI:** 10.3389/fimmu.2021.633540

**Published:** 2021-07-06

**Authors:** Daniella Bianchi Reis Insuela, Maximiliano Ruben Ferrero, Cassiano Felippe Gonçalves-de-Albuquerque, Amanda da Silva Chaves, Adriano Yagho Oliveira da Silva, Hugo Caire Castro-Faria-Neto, Rafael Loureiro Simões, Thereza Christina Barja-Fidalgo, Patricia Machado Rodrigues e Silva, Marco Aurélio Martins, Adriana Ribeiro Silva, Vinicius Frias Carvalho

**Affiliations:** ^1^ Laboratory of Inflammation, Oswaldo Cruz Institute, Oswaldo Cruz Foundation (FIOCRUZ), Rio de Janeiro, Brazil; ^2^ Laboratory of Immunopharmacology, Oswaldo Cruz Institute, Oswaldo Cruz Foundation (FIOCRUZ), Rio de Janeiro, Brazil; ^3^ Laboratory of Immunopharmacology, Biomedical Institute, Federal University of the State of Rio de Janeiro, Rio de Janeiro, Brazil; ^4^ Laboratory of Inflammation, National Institute of Science and Technology on Neuroimmunomodulation (INCT-NIM), Rio de Janeiro, Brazil; ^5^ Laboratory of Cellular and Molecular Pharmacology, Biology Institute, State University of Rio de Janeiro, Rio de Janeiro, Brazil

**Keywords:** cAMP, diabetes, glucagon, neutrophil, sepsis

## Abstract

Sepsis is one of the most common comorbidities observed in diabetic patients, associated with a deficient innate immune response. Recently, we have shown that glucagon possesses anti-inflammatory properties. In this study, we investigated if hyperglucagonemia triggered by diabetes might reduce the migration of neutrophils, increasing sepsis susceptibility. 21 days after diabetes induction by intravenous injection of alloxan, we induced moderate sepsis in Swiss-Webster mice through cecum ligation and puncture (CLP). The glucagon receptor (GcgR) antagonist des-his1-[Glu9]-glucagon amide was injected intraperitoneally 24h and 1h before CLP. We also tested the effect of glucagon on CXCL1/KC-induced neutrophil migration to the peritoneal cavity in mice. Neutrophil chemotaxis *in vitro* was tested using transwell plates, and the expression of total PKA and phospho-PKA was evaluated by western blot. GcgR antagonist restored neutrophil migration, reduced CFU numbers in the peritoneal cavity and improved survival rate of diabetic mice after CLP procedure, however, the treatment did no alter hyperglycemia, CXCL1/KC plasma levels and blood neutrophilia. In addition, glucagon inhibited CXCL1/KC-induced neutrophil migration to the peritoneal cavity of non-diabetic mice. Glucagon also decreased the chemotaxis of neutrophils triggered by CXCL1/KC, PAF, or fMLP *in vitro*. The inhibitory action of glucagon occurred in parallel with the reduction of CXCL1/KC-induced actin polymerization in neutrophils *in vitro*, but not CD11a and CD11b translocation to cell surface. The suppressor effect of glucagon on CXCL1/KC-induced neutrophil chemotaxis *in vitro* was reversed by pre-treatment with GcgR antagonist and adenylyl cyclase or PKA inhibitors. Glucagon also increased PKA phosphorylation directly in neutrophils *in vitro*. Furthermore, glucagon impaired zymosan-A-induced ROS production by neutrophils *in vitro*. Human neutrophil chemotaxis and adherence to endothelial cells *in vitro* were inhibited by glucagon treatment. According to our results, this inhibition was independent of CD11a and CD11b translocation to neutrophil surface or neutrophil release of CXCL8/IL-8. Altogether, our results suggest that glucagon may be involved in the reduction of neutrophil migration and increased susceptibility to sepsis in diabetic mice. This work collaborates with better understanding of the increased susceptibility and worsening of sepsis in diabetics, which can contribute to the development of new effective therapeutic strategies for diabetic septic patients.

## Introduction

Diabetes is frequently associated with the development and worsening of sepsis, with about 22% of septic patients presenting a medical history of diabetes (1, 2). Although the high risk of infections observed in diabetic patients has been linked with defects in the innate and adaptive immune system (3), the mechanisms behind immune deficiency in diabetes are still unknown. It is well established that the major changes in innate immunity of diabetes affect neutrophils, including decreased migration, chemotaxis, phagocytosis, and microbicide activity (4–6). In addition, it was shown that the greater severity of sepsis in type 1 diabetic mice is related to reduce neutrophil migration to the focus of infection (7, 8).

Type 1 diabetes is associated with increased circulating levels of glucagon (9). Furthermore, the severity, organ dysfunction, and mortality were seen in patients with severe sepsis who have a close relationship with high plasma glucagon levels (10), suggesting an association between glucagon overproduction and diabetes immune deficiency. We previously demonstrated that glucagon reduced lipopolysaccharide (LPS)-induced neutrophil migration to airways, increasing the evidence of its anti-inflammatory performance (11). Glucagon is a stimulatory hormone which acts through a 7TM receptor coupled to a Gs protein, inducing an increase in the intracellular levels of cyclic adenosine monophosphate (cAMP) with consequent stimulation of the protein kinase A (PKA)/cAMP response element-binding (CREB) signaling pathway (12).

In neutrophils, the increase of intracellular cAMP levels induced by phosphodiesterase (PDE)-4 inhibitors are associated with reduced recruitment *in vivo* and chemotaxis *in vitro* (13). Inhibition of PKA activity restores LPS-induced neutrophil migration despite the presence of PDE-4 inhibitors (14). Since glucagon presents anti-inflammatory and immunomodulatory effects on neutrophils (15), this work aimed to evaluate the role of glucagon on the impaired neutrophil migration observed in diabetic animals submitted to cecum ligation and puncture (CLP)-induced sepsis. Furthermore, we evaluated the impact of the reestablishment of neutrophil migration by GcgR antagonist in diabetic mice after CLP on bacterial clearance. We believe that this knowledge contributed to a better understanding of the role of glucagon on the increased susceptibility and worsening of sepsis in diabetes, which may benefit future investigations of therapeutic strategies for septic diabetic patients.

## Material and Methods

### Chemicals

Alloxan, Glucagon, Des-his1-[Glu9]-glucagon amide, Rolipram, Percoll, RPMI medium, Hank’s balanced salt solution without Ca^+2^ or Mg^+2^ (HBSS), mouse CXCL1/KC, human CXCL8/IL-8, N-formyl methionyl-leucyl-phenylalanine (fMLP), H-89, SQ 22536, DMSO, LPS, Penicillin, Zymosan A and Streptomycin were purchased from Sigma Chemical Co (St. Louis, MO, USA). Ficoll-Paque PLUS (density 1.077 g/mL) was purchased from GE Healthcare Bio-Sciences (Pittsburgh, PA, USA), platelet-activating factor (PAF) was purchased from Calbiochem (San Diego, CA, USA), and fetal bovine serum (FBS) was purchased from Gibco (Albuquerque, NM, USA). Meropenem was purchased from ABL – Antibióticos do Brasil (São Paulo, SP, Brazil) and Luminol was donated by Prof. Margareth Queiroz from Oswaldo Cruz Institute/Fiocruz (Rio de Janeiro, RJ, Brazil). All the solutions were freshly prepared immediately before use.

### Human Subjects

Peripheral blood samples were obtained from healthy volunteers of both sexes (50% female and 50% male) and aged 25 to 30 years. Donors who reported any kind of infection, inflammation, and/or use of anti-inflammatory or antibiotic medication were excluded from the work.

### Animals

Male Swiss-Webster mice (4-6 weeks-old; weighing 20-25 g) were obtained from the Oswaldo Cruz Foundation breeding colony. Mice were housed in groups of five in a temperature- humidity- and light-controlled (12h light: 12h darkness cycle) colony room. Mice were given *ad libitum* access to food and water. All animal procedures were performed in the laboratory beginning between 8:00 and 9:00 a.m. Animals euthanasia was performed with ketamine (300 mg/Kg, i.p.) (Cristália, São Paulo, SP, Brazil) and xylazine (100 mg/Kg, i.p) (Syntec, Santana de Parnaíba, SP, Brazil) as previously reported (16). Details of animal’s welfare evaluation are described in *Material and Methods* section of **Supplementary Material (SM)**.

### Diabetes Induction

Diabetes was induced by a single intravenous injection of alloxan monohydrate (65 mg/kg) (17), diluted with sterile saline (0.9% NaCl), into 12-h-fasted mice supplied with water *ad libitum*. Mice injected with vehicle and submitted to similar experimental conditions were used as negative controls. Blood glycemia was determined using a glucose monitor (Johnson & Johnson, CA, USA) in samples obtained from the tail vein. After three days of alloxan injection, mice with blood glucose levels below 11 mmol/l were excluded from further experiments.

### Hormones Evaluation

Sixteen mice were randomly assigned into 2 experimental groups: non-diabetic mice (n = 8) and Diabetic mice (n = 8). Twenty-one days after diabetes induction, animals were killed, and blood was immediately collected from the abdominal aorta in vials containing heparin (40 U/mL) for glucagon and insulin quantification. Plasma was obtained after sample centrifugation for 10 min at 1000 ×g. Glucagon and insulin levels were quantified by radioimmunoassay following manufacturer’s guidelines (MP Biomedicals, Solon, OH, USA), using a gamma counter (ICN Isomedic 4/600 HE; ICN Biomedicals Inc., Costa Mesa, CA, USA).

### Surgical Procedure, CLP Model, and Treatments

Twenty-one days after alloxan administration, sepsis was induced by CLP as previously described (18). Briefly, mice were anesthetized with an intraperitoneal injection of ketamine (100 mg/kg, Cristália) and xylazine (10 mg/kg, Syntec) 10–15 min prior to surgery. CLP was performed by two punctures in the ileocecal valve and cecum using a 21-gauge needle to induce sepsis. A small amount of fecal material was extruded from the holes before reinsertion of the cecum in the abdominal cavity. Sham animals were submitted to laparotomy without cecum puncture. Immediately after surgery, the mice received subcutaneous sterile isotonic saline (1 mL) for fluid resuscitation, and 6 h after, the antibiotic meropenem (10 mg/kg) was administrated intraperitoneally (18). Diabetic mice were treated with GcgR antagonist (des-his1-[Glu9]-glucagon amide; 0.3 or 1 mg/kg) (19) by intraperitoneal injection 24h and 1h before CLP. Control mice received an equal volume of vehicle (sterile saline 0.9%). Forty-eight mice were randomly assigned into 6 experimental groups: Non-diabetic sham mice (n = 8); Diabetic sham mice (n = 8); Non-diabetic CLP mice (n = 7); Diabetic CLP mice (n = 8); Diabetic CLP mice treated with GcgR antagonist 0.3 mg/kg (n = 8); Diabetic CLP mice treated with GcgR antagonist 1 mg/kg (n = 9), and the analysis of glycemia, total and differential leukocytes, levels of cytokines and numbers of colony-forming units (CFU) in peritoneal cavity were performed 3h after surgical procedure. In another experiment, seventeen mice were randomly assigned into 4 experimental groups: non-diabetic sham mice (n = 4); Non-diabetic CLP mice (n = 5); Diabetic CLP mice (n = 4); Diabetic CLP mice treated with GcgR antagonist 1 mg/kg (n = 4). The analysis of neutrophils in the blood was performed 3h after surgical procedure, while the evaluation of CXCL1/KC chemokine in plasma was carried out 20h after CLP. For survival rate evaluation, 47 mice were randomly assigned into 5 experimental groups: non-diabetic sham mice (n = 10); Diabetic sham mice (n = 7); Non-diabetic CLP mice (n = 10); Diabetic CLP mice (n = 10); Diabetic CLP mice treated with GcgR antagonist 1 mg/kg (n = 10), and the percentage of live animals were determined from 0 to 24h after CLP.

### Neutrophil Migration to the Peritoneal Cavity

Fourteen healthy mice were randomly assigned in 3 experimental groups: Control group mice (n = 4); CXCL1/KC-stimulated mice (n = 5); Glucagon treated and CXCL1/KC-stimulated mice (n = 5). Animals were pretreated with glucagon (1 µg/Kg, i.p.) (11) or its vehicle (sterile saline 0.9%) 1h before CXCL1/KC injection (200 ng/Cavity, i.p.) and the analysis was performed 3h after stimulation.

### Leukocyte Recovery From the Peritoneal Cavity

Cells were recovered from the peritoneal cavity through lavage with 3 mL of sterile saline in the laminar flow cabinet. Total leukocyte counts were performed in Neubauer chambers using optical microscopy (BX40, Olympus, Center Valley, PA, USA), after diluting the samples (40X) in Turk solution (2% acetic acid). Differential cell counts were carried out on panoptic-stained (Laborclin, Nova Iguaçu, RJ, Brazil) cytospin preparations under the oil immersion objective to determine the percentage of mononuclear cells and neutrophils (18).

### Determination of CFU

The number of CFU in the peritoneal lavage was determined by diluting the samples 10 or 100 times, seeding on culture plates, and incubating under aerobic and sterile conditions on Difco tryptic soy agar (BD Biosciences PharMingen, San Diego, CA, USA) for 24h at 37°C. The number of bacteria colonies were counted and expressed as CFU/cavity (18).

### Neutrophil Evaluation From Blood

The blood was obtained from the tail vein of mice. Neutrophil count was performed in blood smears stained with May-Grunwald–Giemsa dye using optical microscopy (BX40, Olympus) under the oil immersion objective (20).

### Cytokine and Chemokine Evaluation

The peritoneal lavage was recovered and centrifuged (1500 x *g*, 10 min, 4°C). Blood was collected from abdominal aorta in tubes containing 100 µL of 3.2% sodium citrate, and plasma was obtained after centrifugation (1000 x *g*, 10 min, 4°C). Then, IL-6, CXCL1/KC, and CXCL2/MIP-2 were quantified from the supernatant using a commercial ELISA kit according to the manufacturer’s instructions (R&D Systems, Minneapolis, MN, USA).

### Isolation of Neutrophils

Bone marrow (BM)-neutrophils were obtained after flushing the femur and tibia of both posterior paws of mice, as previously described (21). Then, neutrophils were isolated using a discontinuous gradient of percoll (72 and 54%) (22). To obtain human neutrophils, 20 mL of blood was collected from healthy volunteers. After sedimentation and lysis of the red cells with a hypotonic solution of NaCl, neutrophils were isolated using Ficollpaque as previously described (23). All samples were stained with Turk dye solution (2% acetic acid) for the measurement of total cells in a Neubauer chamber using a light microscope (BX40; Olympus). Both cell preparations consisted of more than 90% of neutrophils purity, which was determined by differential cell counts in cytospin smears, stained with May-Grünwald Giemsa method, using a light microscope (BX40, Olympus).

### Viability Assay of Neutrophils *In Vitro*

Neutrophils obtained from humans’ blood or mice BM (1x10^5^ cells/well) were incubated in RPMI medium without FBS and treated with PDE-4 inhibitor rolipram (5 μM), glucagon (0.3-3 μM), or vehicle (DMSO 0.1% or medium, respectively) for 3h *in vitro* at 37°C in a 5% CO2 atmosphere. For the evaluation of neutrophils viability, aliquots of cell suspensions were mixed with trypan blue solution (0.2%) and were counted using a light microscope (BX40, Olympus). Neutrophils that presented blue color in their interior were considered non-viable. The cells presented at least 85% or 95% of viability in all groups studied for neutrophils obtained from mice and humans, respectively.

### Chemotaxis of Neutrophils *In Vitro*

Neutrophils obtained from mice BM or humans’ blood were labeled with calcein-AM (5 μM) for 30 min. After washing with HBSS, neutrophils were pretreated with glucagon (0.03-3 μM), rolipram (5 μM), or vehicle (medium or DMSO 0.1%, respectively) for 30 min or 1h for murine and human cells, respectively, *in vitro* at 37°C in a 5% CO2 atmosphere. Next, chemotaxis assay of murine neutrophils (2x10^5^ cells/well) was performed in a 96 well Neuroprobe ChemoTx^®^ 101-5 chemotaxis plate (Neuro Probe, Gaithersburg, MA, USA), using chemotactic stimuli CXCL1/KC (25 nM), fMLP (1 μM), PAF (20 μM) or medium for 40 min *in vitro*. After that period, the lower compartment of the chemotaxis plate was read in a plate reader (SpectraMax M5, Molecular Devices, San Jose, CA, USA) at an excitation wavelength of 485nm and emission wavelength of 530nm (24). The number of cells that migrated was obtained by comparison with a standard cell curve. In some experiments, murine neutrophils were incubated with glucagon receptor antagonist (des-His1-[Glu9] glucagon amide; 3 μM), adenylyl cyclase inhibitor (SQ 22536; 1 or 10 μM), or PKA inhibitor (H-89; 1 or 10 μM) 30 min before glucagon treatment *in vitro*. In the case of neutrophils obtained from human blood, chemotaxis was induced by CXCL8/IL-8 (12 nM) or medium for 2h *in vitro* at 37°C in a 5% CO2 atmosphere (24). The results were expressed as a percentage of cells that migrated compared to the average percentage of migration in the group of cells pretreated with vehicle and stimulated with CXCL1/KC, fMLP, PAF or CXCL8/IL-8 *in vitro*, which was attributed with a value of 100% migration.

### Evaluation of GcgR, CD11a and CD11b Expression on Neutrophils Surface by Flow Cytometry

Blood neutrophils from non-diabetic or diabetic mice were stained with monoclonal antibody anti-Ly6G (clone 1A8) (FITC 1:400) (Thermo Fisher Scientific, Swedesboro, NJ, USA), a specific marker for neutrophils (25), and primary polyclonal rabbit anti-GcgR antibody (1:100) (Santa Cruz Biotechnology, Santa Cruz, CA, USA) for 30 min, washed with PBS 1X and then incubated with secondary polyclonal anti-rabbit-Alexa 647 (1:100) antibody (Thermo Fisher Scientific) for 30 min to evaluate GcgR expression in Ly6G^+^ cells. For evaluation of GcgR expression in human neutrophils, first these cells were isolated from human blood using Ficollpaque and then labeled with polyclonal antibody anti-GcgR (PE 1:20) (Bioss Antibodies, Woburn, MA, USA. In another set of experiments, isolated neutrophils (5 x 10^5^ cells/well) were treated with glucagon (3 μM) or rolipram (5 μM) for 30 or 40 min for BM-murine and human cells, respectively, *in vitro*. Next, murine BM-neutrophils were activated with CXCL1/KC (10 nM) for 40 min, while human neutrophils were stimulated with CXCL8/IL-8 (12 nM) for 1h *in vitro*. Then, the cells were labeled using monoclonal antibodies anti-Ly6G (clone 1A8) (FITC 1:400), anti-CD11a (PE 1:100), and anti-CD11b (PECY7 1:200) (Thermo Fisher Scientific) for 30 min. The results of CD11a and CD11b translocation to neutrophils surface were represented as a percentage of median fluorescence intensity (MFI) values compared to a 100% baseline MFI values attributed to the group of cells pretreated with vehicle and stimulated with CXCL1/KC, for murine, or CXCL8/IL-8, for human cells, *in vitro*. In all experiments, before incubation with the respective antibodies, all the cells were exposed to sheep serum for 15 min at 4°C to block nonspecific binding, and then washed with PBS 1X. In addition, as control of nonspecific binding, we used isotype-matched antibodies, or the primary polyclonal rabbit antibody anti-GcgR (Santa Cruz Biotechnology) was omitted. All the results were acquired by flow cytometry (FACSCalibur; BD Biosciences PharMingen) and analyzed using FlowJo software (Tree Star, Inc., Ashland, OR, USA). Gating strategies for the identification of neutrophils in these experiments are shown in **SM Figures S1**, **S3**, **S4**, **S7**–**S9**. The performance of neutrophil isolation with Ficollpaque or Percoll was evaluated by differential cell counts in cytospin smears, stained with May-Grünwald Giemsa method, using a light microscope (BX40, Olympus). All cell preparations consisted of more than 90% of neutrophils purity.

### F-Actin Expression in Neutrophils *In Vitro*

Mice BM-neutrophils (2x10^5^ cells/well) were pretreated with glucagon (3 μM), rolipram (5 μM) or vehicle (medium or DMSO 0.1%, respectively) for 30 min and, then, stimulated with CXCL1/KC (10 nM) or medium for 40 min *in vitro* at 37°C in a 5% CO2 atmosphere. Neutrophil slides were prepared by cytospin centrifuge, F-actin was stained with TRITC-labeled phalloidin (Sigma), and actin polymerization was evaluated by fluorescence microscopy, as previously described (26). Briefly, actin polymerization was accessed through the analysis of the mean fluorescence intensity in randomly chosen fields of each slide. Images were acquired by optical microscopy using the same parameters settings of the control group (time of exposure, brightness, contrast, and sharpness), and values of mean fluorescence intensity were obtained using Adobe Photoshop^®^ software. To achieve a more accurate quantification, mean fluorescence intensity was determined to discount the background of each field, and the results normalized by the number of cells per field counted by DAPI fluorescence. The results were expressed as a percentage of F-actin expression compared to a 100% baseline for cells pre-treated with vehicle and stimulated with medium *in vitro*.

### Evaluation of GcgR Expression and PKA Phosphorylation (pPKA) in Neutrophils by Western Blot

Neutrophils were isolated from mice BM for evaluation of GcgR expression. Then, isolated BM-neutrophils (1x10^6^ cells/well) were treated with glucagon (3 μM) or vehicle (medium) at several time intervals (5, 15, and 30 min) *in vitro* at 37°C in a 5% CO2 atmosphere. The cells were homogenized in cold RIPA buffer containing a cocktail for protease and phosphatase inhibitors (1:100; Thermo Fisher Scientific). Cell supernatant was collected, denatured and the total proteins were quantified by using the BCA method (Sigma). For western blot analysis, equal amounts of proteins were resolved on SDS–polyacrylamide gel electrophoresis and subsequently transferred onto nitrocellulose membranes, as previously described (27). After blocking, membranes were incubated overnight at 4°C with specific mouse monoclonal anti-PKA (1:500; Santa Cruz Biotechnology), rabbit polyclonal anti-pPKA (1:500; Santa Cruz Biotechnology), rabbit polyclonal anti-GcgR (1:300; Santa Cruz Biotechnology) or rabbit monoclonal anti-β-actin (1:1000; Cell Signaling Technology, Danvers, MA, USA) antibodies. After washing, membranes were incubated with anti-mouse or anti-rabbit HRP-secondary antibody (1:10000; R&D System) for 1h at room temperature. Specific protein bands were detected using ECL (SuperSignal West Dura, Thermo Fisher Scientific). Band intensity was quantified by densitometry using ImageJ software (NIH, Bethesda, MD, USA). The results of PKA phosphorylation were expressed as a percentage of ratio between the expressions of pPKA/PKA compared to a 100% baseline attributed to cells treated with vehicle *in vitro*.

### Analysis of ROS Production by Neutrophils *In Vitro*

ROS was measured through the kinetics of the chemiluminescence of luminol (28) after stimulation with zymosan A. Briefly, BM-neutrophils isolated from mice (3x10^5^ cells/well) were pretreated with glucagon (0.3-3 μM), rolipram (5 μM) or vehicle (medium or DMSO 0.1%, respectively) for 30 min *in vitro* at 37°C in a 5% CO2 atmosphere. Then, the cells were stimulated with zymosan A (0.1 mg/mL) opsonized with mouse serum, in the presence of luminol (1 mM). The chemiluminescence reaction was monitored by a plate reader (SpectraMax M5, Molecular Devices) for 1h at 37°C. ROS production was represented as the area under the curve (AUC) of the time-course.

### Assessment of Neutrophil Adhesion to Endothelial Cells

Human umbilical vein endothelial cells (HUVECs) were cultured in RPMI medium supplemented with penicillin 1 × 10^6^ U/mL, 0.2 g/mL streptomycin, and 10% FBS at 37°C in a 5% CO2 atmosphere. Cells were grown to 90% confluence in 96-wells plates and were stimulated with LPS (1 µg/mL; *E. coli*, serotype 026: B6) for 4 h prior to the adhesion assay *in vitro*. After stimulation, the cells were washed with PBS 1X. In parallel, neutrophils were isolated from human blood (5x10^5^ cells/well) and labeled with calcein-AM (5 μM) for 30 min. After washing with HBSS, these cells were pretreated with glucagon (0.3-3 μM) or vehicle (medium) for 40 min *in vitro* at 37°C in a 5% CO2 atmosphere. Next, neutrophils were stimulated with CXCL8/IL-8 (12 nM) or vehicle (medium) for 1h *in vitro*, and then the wells were washed with HBSS.

For adhesion assay, labeled-stimulated-neutrophils were incubated (2 x 10^5^ cells/well) to wells containing LPS-provoked-HUVECs for 1h at 37°C in a 5% CO2 atmosphere. Non-adherent cells were then removed by gentle aspiration and washing with PBS 1X, and the number of adherent neutrophils was determined with a fluorescence plate reader (SpectraMax M5, Molecular Devices) using an excitation wavelength of 485 nm and an emission wavelength of 538 nm (29). For each condition, duplicate wells were tested, and values are shown as the percent of added neutrophils remaining adherent to the HUVEC monolayers compared to a 100% baseline attributed to adherent neutrophils that were pre-treated with vehicle and stimulated with CXCL8/IL-8 *in vitro*.

### Evaluation of CXCL8/IL-8 Released by Neutrophils *In Vitro*

Neutrophils isolated from human blood (5x10^5^ cells/well) were treated with glucagon (0.3-3 μM) for 1h and, then, stimulated with LPS (10 ng/mL) (30) for 2h *in vitro* at 37°C in a 5% CO2 atmosphere. Next, the levels of the chemokine CXCL8/IL-8 were quantified from the supernatant, using a commercial ELISA kit (R&D Systems) according to the manufacturer’s instructions. The results are expressed as the percent of IL-8 release compared to a 100% baseline for cells pre-treated with vehicle and stimulated with CXCL8/IL-8 *in vitro*.

### Statistical Analysis

The data are reported as the mean ± standard error of the mean (SEM). All data were evaluated to ensure normal distribution and statistically analyzed by one-way ANOVA, followed by a Student-Newman–Keuls *post hoc* test. In the case of the survival rate, the χ^2^-test (Fisher’s exact test) was used. All statistical analysis was performed with GraphPad Prism 8 software (La Jolla, CA, USA). Probability values (*P*) of 0.05 or less were considered significant.

## Results

### Hyperglucagonemia Impairs CLP-Induced Neutrophil Migration and Bacterial Clearance in the Peritoneal Cavity of Diabetic Mice

Twenty-one days after alloxan-induced diabetes, mice presented increased blood glucose levels with a simultaneous reduction of body weight and plasma insulin levels (**Table 1**), indicating a diabetic state. Furthermore, we noted an increase in the plasma levels of glucagon in our model of diabetes (**Table 1**). Before the induction of CLP, diabetic mice treated with 1 mg/kg GcgR antagonist showed a significant reduction, although partial, of the blood glucose levels (26 ± 3.3 mmol/L; mean ± SEM; n = 9) comparing to untreated diabetic mice (32 ± 0.6 mmol/L; mean ± SEM, n=8; *P* < 0.05). The values of circulating glucose of untreated non-diabetic mice were 8 ± 0.3 mmol/L (mean ± SEM, n=9; *P* < 0.05 compared to untreated diabetic mice).

**Table 1:** Alloxan-induced diabetes causes hyperglycemia, loss of body weight, hypoinsulinemia, and hyperglucagonemia.

Group	Glycaemia (mmol/l)	Body Weight (g)	Plasma Insulin (µU/ml)	Plasma Glucagon (ρg/ml)
Non-diabetic	6 ± 0.5	36 ± 1	36 ± 7	48 ± 5
Diabetic	22 ± 0.8^+^	27 ± 1^+^	8 ± 2^+^	118 ± 31^+^

Diabetes was induced by a single intravenous injection of alloxan (65 mg/kg), and the analyses performed 21 days after diabetes induction. Values represent the mean ± S.E.M. of 8 animals. ^+^P < 0.05 compared to non-diabetic mice.

**Figure 1:**
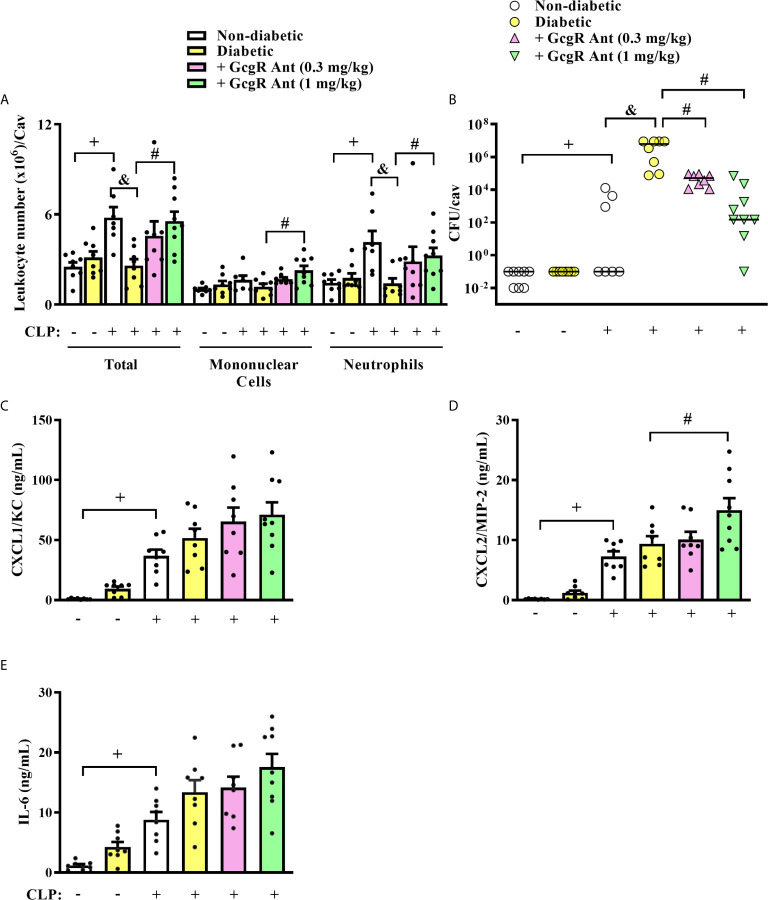
GcgR antagonist restores neutrophil migration and inhibits CFU formation but does not alter chemokines and IL-6 production in the peritoneal cavity of diabetic mice after sepsis induction. CLP was performed 21 days after diabetes induction, and the treatment with GcgR antagonist was carried out 24h and 1h before CLP. **(A)** Analysis of total and differential leukocytes. **(B)** Bacterial load evaluation. **(C–E)** Peritoneal levels of CXCL1/KC, CXCL2/MIP-2, and IL-6, respectively. All the analysis were realized 3h after CLP. Each value represents the mean ± S.E.M. For **(A, B)**, each sample value was obtained in blinded and randomized counting. The statistical analysis was performed by one-way ANOVA, followed by Newman–Keuls–Student’s t-test. Results are representative of two independent assays. ^+^ *P* < 0.05 compared to sham non-diabetic mice. ^&^ *P* < 0.05 compared to CLP non-diabetic mice. ^#^ *P* < 0.05 compared to CLP diabetic mice. CFU, Colony-forming unit; CLP, Cecum ligation and puncture; GcgR Ant, GcgR Antagonist.

Diabetic mice subjected to CLP showed decreased accumulation of total leukocytes, mainly neutrophils, into the peritoneal cavity compared to non-diabetic mice subjected to CLP (**Figure 1A**). This failure in neutrophil migration observed in diabetic animals was accompanied by an increase in the CFU counts in the peritoneal fluid (**Figure 1B**). Treatment of diabetic mice with GcgR antagonist at a dose of 1 mg/kg, but not at 0.3 mg/kg restored CLP-induced accumulation of total leukocytes and neutrophils in the peritoneal cavity (**Figure 1A**). Although CLP did not induce mononuclear cells accumulation in the peritoneal cavity of non-diabetic nor diabetic mice, the GcgR antagonist significantly increased the number of mononuclear cells at a dose of 1 mg/kg (**Figure 1A**). Furthermore, GcgR antagonism improved bacterial clearance in the peritoneal cavity of diabetic mice subjected to CLP in a dose-dependent manner as CFU counts were consistently decreased by GcgR treatment (**Figure 1B**).

CLP increased similarly the levels of CXCL1/KC, CXCL2/MIP-2, and IL-6 in the peritoneal cavity of non-diabetic and diabetic mice as compared to non-diabetic sham-mice (**Figures 1C–E**, respectively). GcgR antagonist at a dose of 1 mg/kg was able to increase the levels of CXCL2/MIP-2 in the peritoneal cavity of diabetic mice as compared to untreated diabetic mice subjected to CLP (**Figure 1D**) but did not interfere with the levels of CXCL1/KC (**Figure 1C**) and IL-6 (**Figure 1E**). GcgR antagonist at 0.3 mg/kg had no effect on cytokine and chemokine levels (**Figures 1C–E**, respectively).

### GcgR Antagonist Improved Survival Rate in Diabetic Mice Submitted to the CLP Procedure, But Did Not Alter Glycemia Nor Systemic Inflammation

**Figure 2:**
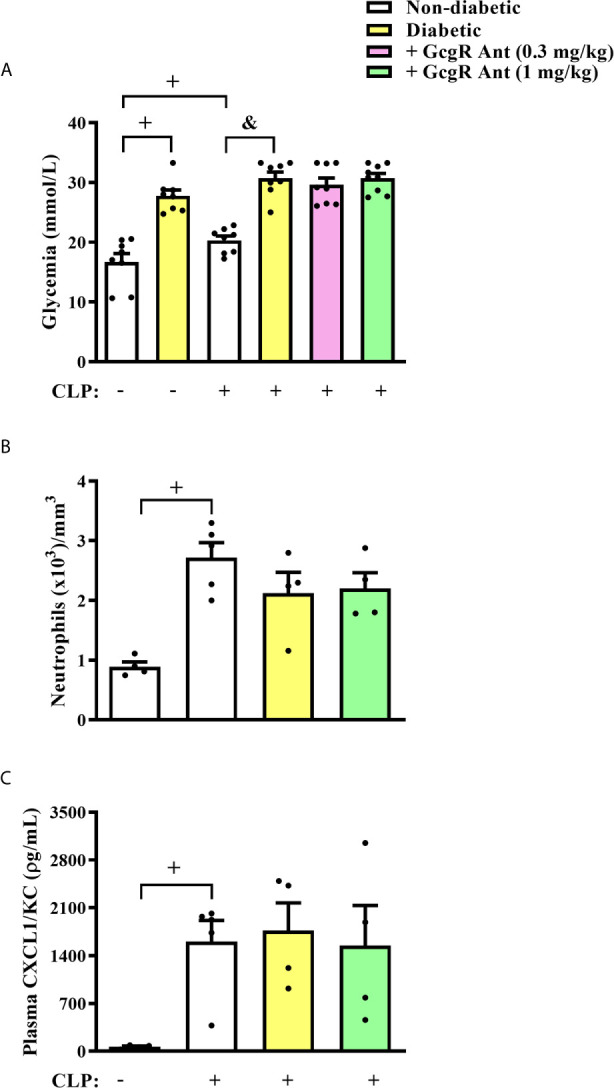
GcgR antagonist did not alter glycemia and systemic inflammation in diabetic mice after CLP induction. CLP was performed 21 days after diabetes induction, and the treatment with GcgR antagonist was carried out 24h and 1h before CLP. **(A)** Glycemia. **(B)** Analysis of blood neutrophil numbers. **(C)** Plasma levels of CXCL1/KC. For **(A, B)** analysis were realized 3h after CLP. For **(C)** analysis was realized 20h after CLP. Each value represents the mean ± S.E.M. For **(B)**, each sample value was obtained in blinded and randomized counting. The statistical analysis was performed by one-way ANOVA, followed by Newman–Keuls–Student’s t-test. Results are representative of one individual assay. ^+^ *P* < 0.05 compared to sham non-diabetic mice. ^&^ *P* < 0.05 compared to CLP non-diabetic mice. CLP, Cecum ligation and puncture; GcgR Ant, GcgR Antagonist.

Three hours after CLP, non-diabetic septic mice showed significantly increased levels of glycemia as compared to non-diabetic sham mice (**Figure 2A**). Diabetic mice subjected to CLP showed increased levels of blood glucose when compared to non-diabetic mice subjected to CLP. None of the doses of the GcgR antagonist interfered with glycemia of diabetic mice subjected to CLP (**Figure 2A**). In addition, we observed an increase in blood neutrophilia (**Figure 2B**) and circulating levels of CXCL1/KC (**Figure 2C**) in non-diabetic subjected to CLP mice when compared to the non-diabetic sham group. Diabetic mice subjected to CLP did not present alteration in blood neutrophilia neither in the circulating levels of CXCL1/KC compared to non-diabetic mice subjected to CLP (**Figures 2B, C**, respectively). In addition, while all non-diabetic mice survived 24h after the CLP procedure, diabetic mice showed only a 50% survival rate (**Table 2**). Altogether, these data confirmed that in our model, sepsis was more severe in diabetic than in non-diabetic mice.

**Table 2:** GcgR antagonist partially improved the survival of diabetic mice subjected to CLP.

Group	Dead/Total	Survival (%)
Sham non-diabetic	0/10	100
CLP non-diabetic	0/10	100
Sham diabetic	0/7	100
CLP diabetic	5/10	50^&^
CLP diabetic + GcgR Ant (1 mg/Kg, i.p.)	3/10	70

CLP was performed 21 days after alloxan-induced-diabetes, and the treatment with GcgR antagonist (1 mg/Kg, i.p.) was carried out 24h and 1h before CLP. The survival rate was evaluated from 0 to 24h after induction of sepsis through CLP. CLP, Cecum ligation and puncture; GcgR Ant, GcgR Antagonist. ^&^ P < 0.05 compared to CLP non-diabetic group.

Treatment of diabetic-CLP mice with 1 mg/kg GcgR antagonist improved the survival rate (**Table 2**) of mice, independently of blood neutrophilia and levels of CXCL-1/KC in plasma, which were not altered by the treatment (**Figure 2**). These results suggest that the control of peritoneum cavity infection by GcgR antagonist is important to improve disease outcomes in diabetic mice subjected to CLP procedure.

### Diabetes Increases the Circulating Ly6G^+^GcgR^+^ Population

**Figure 3:**
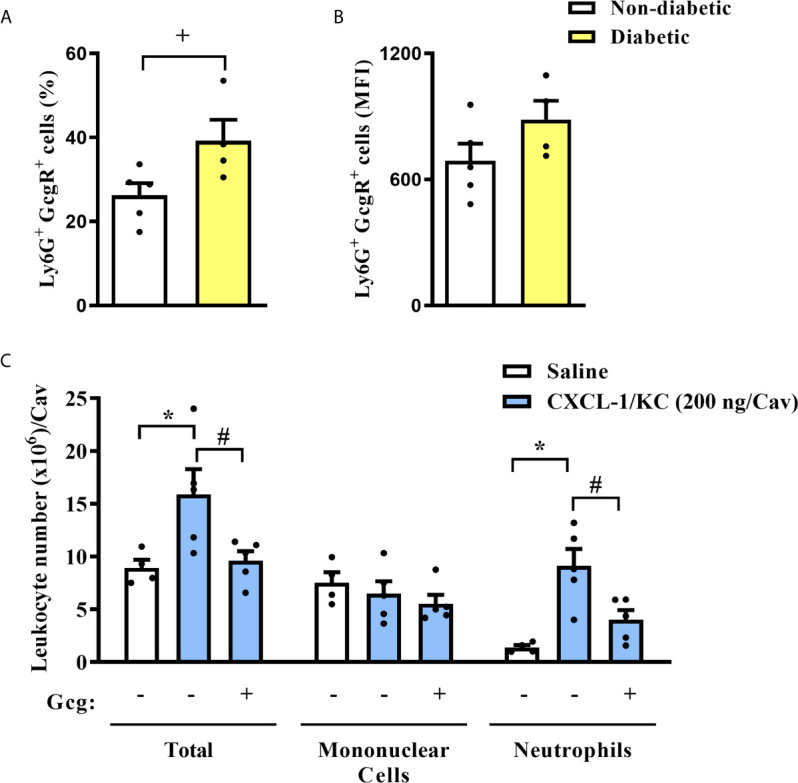
Diabetes induces an increase in the neutrophils GcgR^+^ proportions, and glucagon inhibits CXCL1/KC-induced neutrophil migration to the peritoneal cavity in *vivo*. **(A)** Percentage of Ly6G^+^ cells that express GcgR obtained from the blood of non-diabetic and diabetic mice. **(B)** MFI of GcgR on Ly6G^+^ cells from the blood of non-diabetic and diabetic mice. For **(A, B)**, blood neutrophils from 5 non-diabetic and 4 diabetic male mice were obtained. **(C)** Analysis of total and differential leukocytes in the peritoneal cavity of mice 3h after challenge with CXCL1/KC. Each sample value was obtained in blinded and randomized measurements. Glucagon (1 µg/kg) was injected i.p. 1h before challenging with the chemokine. Each value represents the mean ± S.E.M. The statistical analysis was performed by one-way ANOVA, followed by Newman–Keuls–Student’s t-test. Results are representative of one individual assay. ^+^ *P* < 0.05 compared to non-diabetic animals. ^*^ *P* < 0.05 compared to saline-challenged mice. ^#^ *P* < 0.05 compared to CXCL1/KC-challenged mice. Gcg, Glucagon.

Analyzing only Ly6G^+^ cells from the blood of mice, we observed that diabetic group had increased proportions of Ly6G^+^ cells expressing GcgR in the blood as compared to non-diabetic group (**Figure 3A**). However, we did not note alterations in the expression rate of GcgR observed by MFI in those Ly6G^+^ cells (**Figure 3B**).

### Glucagon Inhibits Neutrophil Migration and ROS Production *In Vitro*

To investigate whether glucagon had a direct impact on neutrophil migration, the chemotactic response was assessed *in vivo* and *in vitro*. First, we challenged mice with an intraperitoneal injection of CXCL1/KC and noted an accumulation of neutrophils in the peritoneal cavity as compared to saline-challenged mice (**Figure 3C**). Mononuclear cell numbers in the peritoneal cavity of mice were not affected by CXCL1/KC. The pre-treatment with glucagon reduced CXCL1/KC-induced neutrophil accumulation in the peritoneal cavity without altering mononuclear cell numbers (**Figure 3C**).

**Figure 4:**
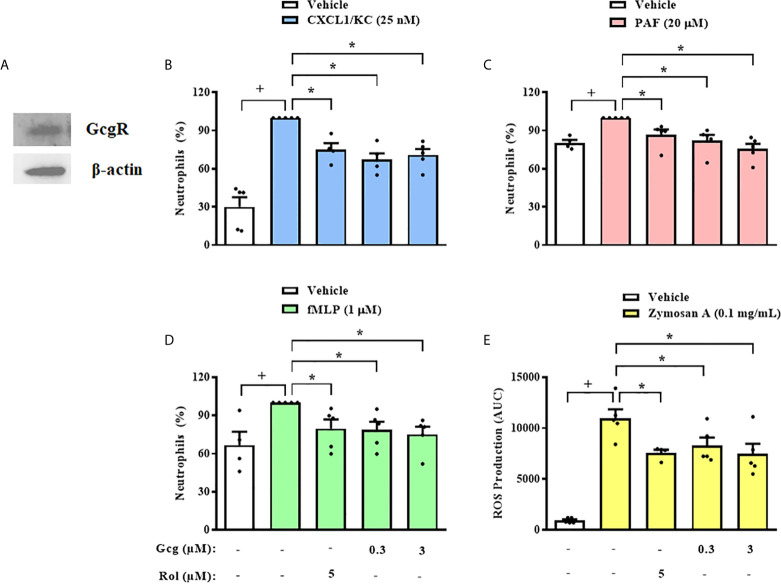
Glucagon inhibits mice neutrophils chemotaxis and ROS production in *vitro*. **(A)** Representative sample of GcgR and β-actin expression in neutrophils obtained from mice BM. The expressions of GcgR and β-actin were determined by western blot. Results are representative of 3 animals. Full-length blots of GcgR and β-actin are reported in **SM Figure S2**. **(B–D)** Effect of glucagon on CXCL1/KC- (25nM), PAF- (20 µM), or fMLP-induced (1 µM) chemotaxis of neutrophils isolated from mice BM in *vitro*, respectively. Cells were pretreated 30 min with glucagon, rolipram or vehicle in *vitro* and then let to migrate for 40 min towards stimulus in a chemotaxis chamber. **(E)** ROS production by BM-neutrophils in *vitro*. Cells were pretreated 30 min with glucagon, rolipram or vehicle in *vitro* and then stimulated with zymosan A (0.1 mg/mL) for 1 h. The vehicles used were DMSO 0.1% or medium to rolipram and glucagon, respectively. Each value represents the mean ± S.E.M. from 5 animals. The statistical analysis was performed by one-way ANOVA, followed by Newman–Keuls–Student’s t-test. Results from **(B–E)** are representative of three individual assays. Results from **(A)** are representative of one individual assay. ^+^ *P* < 0.05 compared to non-stimulated cells. ^*^ *P* < 0.05 compared to stimulated cells. Gcg, Glucagon; Rol, Rolipram.

Next, we obtained neutrophils from mice BM and we observed that these cells also express GcgR (**Figure 4A**). Then, we quantified migration of mice BM-neutrophils towards several chemoattractants, including CXCL1/KC (**Figure 4B**), PAF (**Figure 4C**), and fMLP (**Figure 4D**) *in vitro*. Rolipram (5 µM) treatment, which was used as a positive control of migration inhibition, effectively inhibited the chemotactic response of neutrophils towards CXCL1/KC, PAF, and fMLP (**Figures 4B–D**, respectively). Glucagon, at concentrations of 0.3 and 3 µM, also inhibited the chemotactic response of neutrophils towards all chemoattractants tested (**Figures 4B–D**, respectively).

In addition to migration, we evaluated whether glucagon has direct action on another important activity of neutrophils, which is the production of ROS. We observed that glucagon (0.3 and 3 µM) and rolipram (5 µM) inhibited ROS production induced by zymosan A by mice BM-neutrophils *in vitro* (**Figure 4E**).

### Glucagon Inhibits CXCL1/KC-Induced Actin Polymerization in Neutrophils *In Vitro*

**Figure 5:**
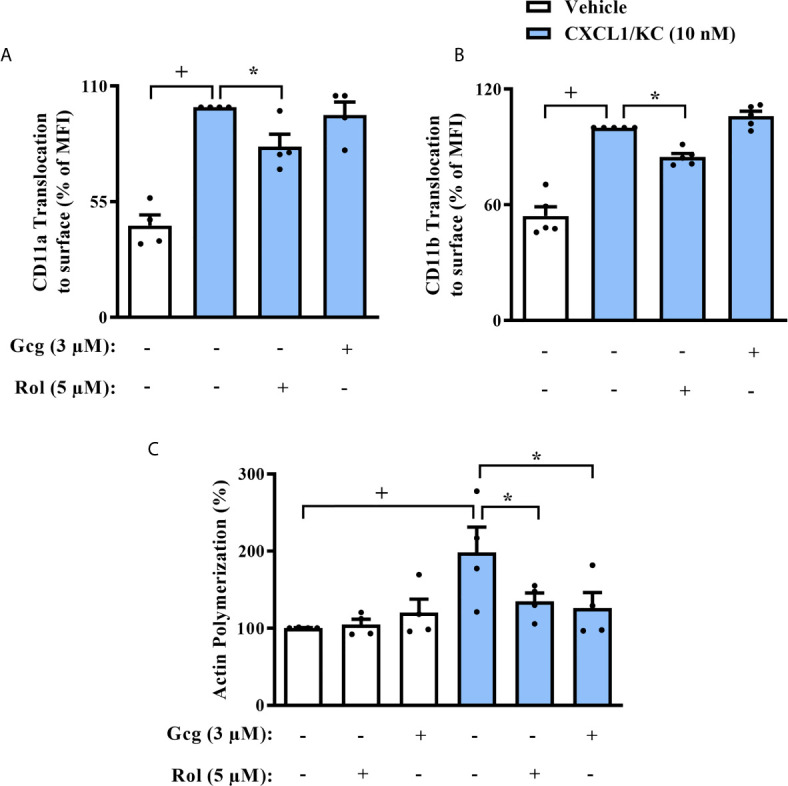
Glucagon inhibits CXCL-1/KC-induced actin polymerization in mice neutrophils in *vitro* but does not alter CD11a nor CD11b translocation to surface. Cells were pre-treated 30 min with glucagon, rolipram or vehicle in *vitro* and then stimulated with CXCL1/KC (10 nM) or medium for 40 min in *vitro*. **(A)** CD11a and **(B)** CD11b translocation to neutrophils surface was represented as the percentage of MFI values that were obtained by FACS. Representative density plots of CD11a and CD11b expression on murine BM-neutrophils surface are reported in **SM Figures S3** and **S4**, respectively. **(C)** Actin polymerization was performed by immunofluorescence with TRITC-labeled phalloidin. Representative images of actin filaments stained with TRITC-phalloidin are reported in **SM Figure S5**. For **(A, C)**, BM-neutrophils from 4 random male mice were obtained. For **(B)**, BM-neutrophils from 5 random male mice were obtained. Each animal sample was equally distributed to all experimental groups. Sample results were obtained by blinded and randomized analysis. The vehicles used were DMSO 0.1% or medium to rolipram and glucagon, respectively. Each value represents the mean ± S.E.M. The statistical analysis was performed by one-way ANOVA followed by Newman–Keuls–Student’s t-test. Results are representative of one individual assay. ^+^ *P* < 0.05 compared to non-stimulated cells. ^*^ *P* < 0.05 compared to stimulated cells. Gcg, Glucagon;. Rol, Rolipram.

In order to elucidate the mechanism by which glucagon inhibits neutrophil chemotaxis, we first evaluated the presence of integrins on the surface of these cells. Glucagon did not change CXCL1/KC-induced translocation of CD11a (**Figure 5A**) nor CD11b (**Figure 5B**) to neutrophils surface *in vitro*, however, rolipram significantly reduced the presence of both integrins in cell surface (**Figures 5A, B**). Actin filaments network dynamic is a crucial process for cell polarization and orientated migration, thus, we analyzed if glucagon could affect chemokine-induced actin polymerization. We noted that similarly to rolipram, glucagon decreased actin polymerization of neutrophils after incubation with CXCL1/KC, without any observed effect on non-stimulated neutrophils (**Figure 5C**).

### Glucagon Inhibits Chemotaxis of Neutrophils *In Vitro* Through Activation of the cAMP-PKA Pathway

**Figure 6:**
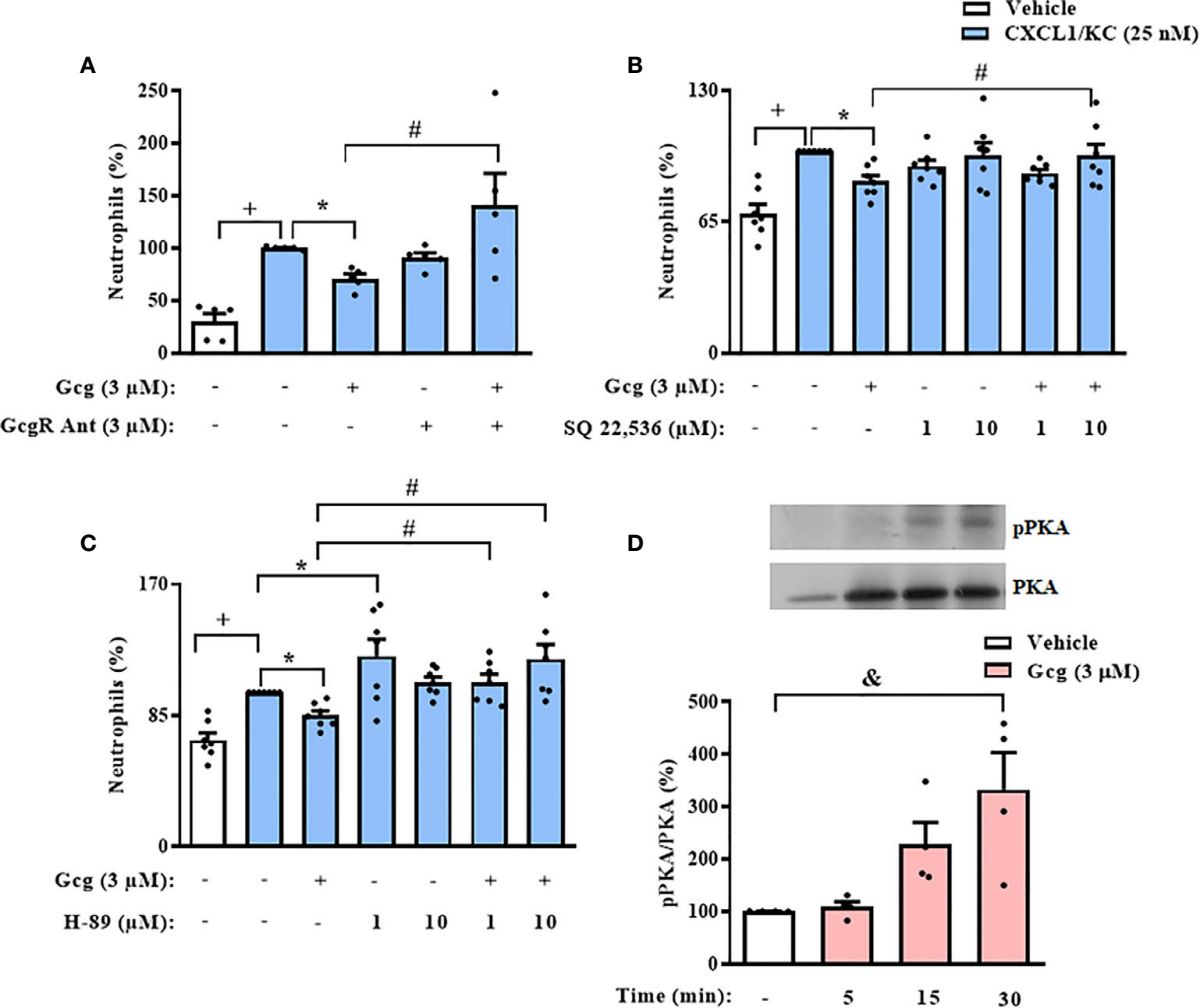
The inhibition of neutrophil chemotaxis by glucagon in *vitro* depends on *the* cAMP-PKA pathway. Cells were pretreated 30 min with GcgR antagonist **(A)**, adenylyl cyclase inhibitor SQ 22,536 **(B)**, or PKA inhibitor H-89 **(C)** and, after this, treated with glucagon for 30 min in *vitro*. Then, cells were let to migrate for 40 min towards CXCL1/KC (25 nM) in a chemotaxis chamber. The vehicles used were DMSO 0.1% to SQ 22,536 or medium to glucagon and H-89. **(D)** Representative samples of pPKA and total PKA expression in the neutrophils of glucagon-treated (3 µM) cells. The expressions of pPKA and total PKA were determined by western blot. Data were normalized to total PKA. Full-length blots of pPKA and total PKA are reported in **Supplementary Figure S6**. Each value represents the mean ± S.E.M. BM-neutrophils obtained from 5 **(A)**, 7 **(B, C)**, and 4 **(D)** random male mice were equally distributed in all experimental groups. The statistical analysis was performed by one-way ANOVA, followed by Newman–Keuls–Student’s t-test. Results from **(A–C)** are representative of two individual assays. Results from **(D)** are representative of one individual assay. ^+++^ *P* < 0.001 compared to non-stimulated cells. ^*^ *P* < 0.05 compared to stimulated cells. ^#^ *P* < 0.05 compared to stimulated cells treated with glucagon. ^&^ *P* < 0.05 compared to non-stimulated cells treated with vehicle. Gcg, Glucagon; GcgR Ant, Glucagon receptor antagonist.

Since we observed that glucagon inhibited neutrophil chemotaxis *in vitro*, we evaluated if this effect depended on the activation of its receptor. We treated neutrophils with GcgR antagonist before glucagon treatment and performed the chemotaxis assays. We showed that glucagon decreased CXCL1/KC-induced chemotaxis of neutrophils *in vitro* and that the GcgR antagonist prevented this effect. GcgR antagonist alone did not affect the chemotaxis of neutrophils triggered by CXCL1/KC (**Figure 6A**).

To investigate the possible signaling pathways involved in the inhibitory effect of glucagon over neutrophil chemotaxis *in vitro*, we pre-incubated these cells with SQ 22,536, an adenylyl cyclase inhibitor, or H-89, a PKA blocker. The pre-treatment with either SQ 22,536 or H-89 prevented the inhibitory action of glucagon on CXCL1/KC-induced chemotaxis of neutrophils (**Figures 6B, C**, respectively); however, neither the adenylyl cyclase inhibitor nor the PKA blocker alone altered the neutrophil migration evoked by CXCL1/KC (**Figures 6B, C**, respectively). Further, we performed western blot analysis to confirm the effect of glucagon over PKA activation in neutrophils. Glucagon induced phosphorylation of PKA in neutrophils 15–30 min after treatment *in vitro* (**Figure 6D**).

### Glucagon Inhibits Chemotaxis and Adhesion of Human Neutrophils *In Vitro* But Does Not Alter the Translocation of CD11a and CD11b

**Figure 7:**
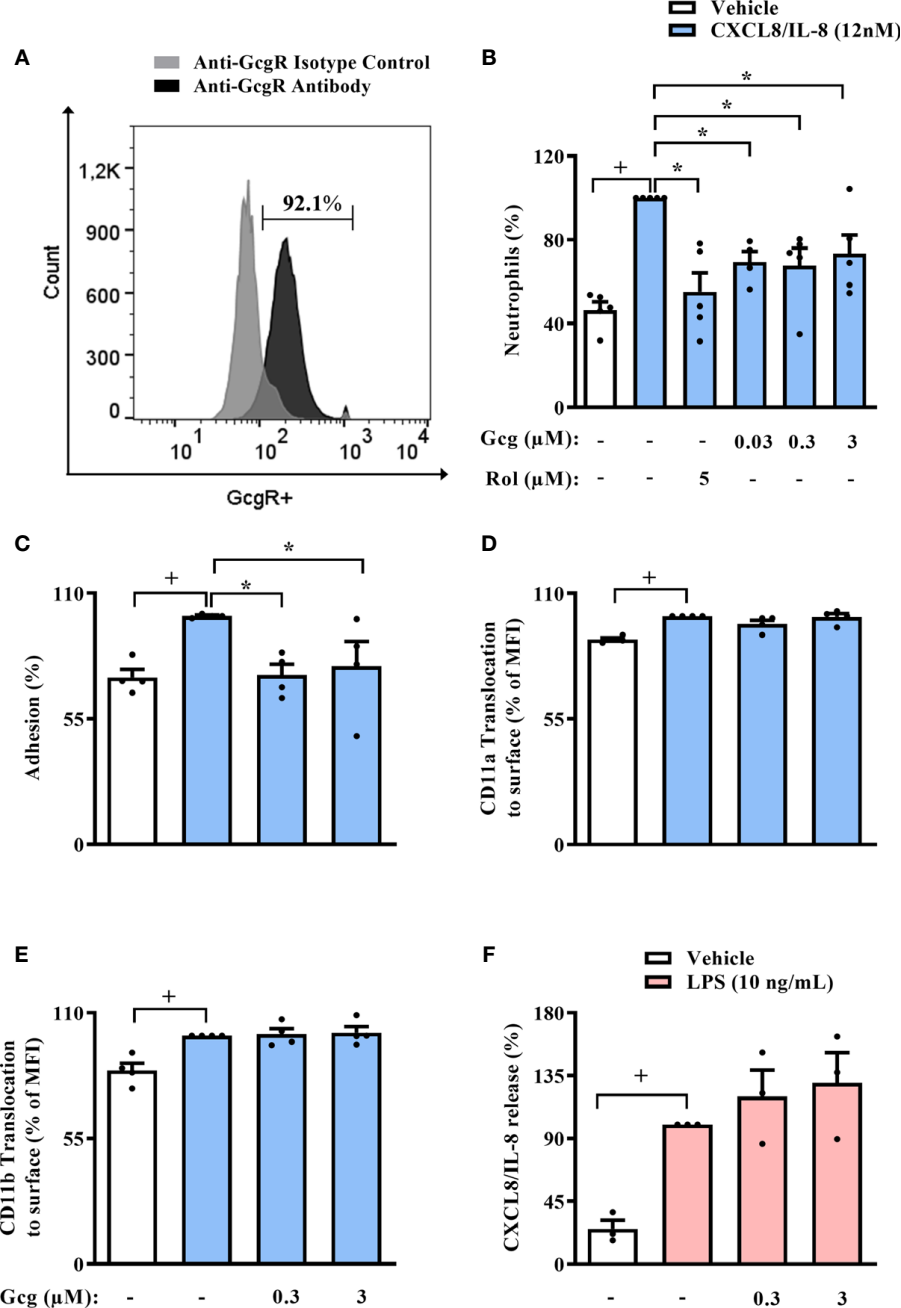
Glucagon inhibits human neutrophil migration and adhesion *in vitro*, without modifying CD11a nor CD11b surface expression and release of CXCL8/IL-8. **(A)** Histogram of GcgR expression on the surface of human neutrophils isolated from healthy individuals. Data are representative of 3 healthy individuals. The gray histogram shows isotype control staining, and the black histogram shows specific staining with polyclonal antibody anti-GcgR (PE). In this assay, 92.1% of neutrophils were GcgR^+^ and MFI value for anti-GcgR antibody was 218, while MFI value for isotype control was 76.1. Representative density plots of GcgR expression and isotype control staining on neutrophils isolated from human blood are reported in **SM Figure S7**. **(B–E)** Effect of glucagon on CXCL8/IL-8-induced (12 nM) chemotaxis; adhesion; and translocation of CD11a and CD11b to surface of neutrophils obtained from human blood *in vitro*, respectively. **(F)** Effect of glucagon on LPS (10 ng/mL)-induced CXCL8/IL-8 release by human neutrophils *in vitro*. For **(B)**, neutrophils from 5 healthy individuals were obtained. Cells were pretreated 1h with glucagon, rolipram or vehicle *in vitro* and then let to migrate for 2h towards CXCL8/IL-8 (12 nM) or medium in a chemotaxis chamber. For **(C)**, neutrophils from 4 healthy individuals were labeled with calcein-AM and pretreated 40 min with glucagon or medium *in vitro*. Then, neutrophils were stimulated with CXCL8/IL-8 (12nM) or medium for 1h *in vitro* and added to previous LPS (1 µg/mL)-activated-HUVECs wells for 1h. Control cells were HUVEC stimulated with LPS and neutrophils incubated with vehicle (medium), while stimulated cells were LPS-provoked HUVEC and CXCL8/IL-8-stimulated neutrophils. The number of adherent neutrophils to HUVECs was determined using a fluorescence plate reader. For **(D, E)**, neutrophils from 4 healthy individuals were obtained. Cells were treated 40 min with glucagon *in vitro* and then stimulated with CXCL8/IL-8 (12 nM) for 1h *in vitro*. Translocation of CD11a and CD11b to cell surface was represented as the percentage of MFI values that were obtained by FACS. Representative density plots of CD11a and CD11b expression on human neutrophils surface are reported in **SM Figures S8**, **S9**, respectively. For **(F)**, neutrophils from 3 healthy individuals were obtained. Cells were pre-treated 1h with glucagon *in vitro* and then stimulated with LPS (10 ng/mL) for 2 h in *vitro*. CXCL8/IL-8 levels were quantified by ELISA. The vehicles used were DMSO 0.1% or medium to rolipram and glucagon, respectively. Each human sample was equally distributed to all experimental groups. Sample results were obtained by blinded and randomized analysis. Each value represents the mean ± S.E.M. The statistical analysis was performed by one-way ANOVA, followed by Newman–Keuls–Student’s t-test. Results from **(A, B)** are representative of two individual assays. Results from **(C–F)** are representative of one individual assay. ^+^ *P* < 0.05 compared to non-stimulated cells. ^*^ *P* < 0.05 compared to stimulated cells. Gcg, Glucagon; Rol, Rolipram.

In order to support the translational relevance of our findings, we first evaluated if human circulating neutrophils express GcgR. By flow cytometry analysis, we showed that approximately 90% of neutrophils isolated from human blood express GcgR on its surface (**Figure 7A**), and the average MFI values of GcgR in human circulating neutrophils was 266. Pre-treatment of neutrophils with glucagon at all concentrations used (0.03-3 µM) or rolipram (5 µM) inhibited chemotaxis towards CXCL8/IL-8 (12 nM) *in vitro* (**Figure 7B**). In addition, glucagon reduced the adhesion of CXCL8/IL-8-activated human neutrophils in HUVEC cells stimulated with LPS *in vitro* (**Figure 7C**). On the other hand, glucagon did not affect the CXCL8/IL-8-induced translocation of both CD11a and CD11b to cell surface (**Figures 7D, E**, respectively), neither CXCL8/IL-8 production stimulated by LPS (**Figure 7F**) *in vitro*, as assessed by flow cytometry and ELISA respectively.

## Discussion

In the present study, we assessed the contribution of glucagon on the increased susceptibility of diabetic mice to sepsis induced by CLP, focusing on the effects of glucagon on neutrophil migration. It is well known that the severity of sepsis is correlated with the impairment of neutrophil migration and function (31). In addition, both diabetic patients and animals present higher susceptibility to bacterial infection (32, 33). In diabetics, the severity of sepsis correlates with decreased neutrophil migration to the site of infection, reduction in neutrophil phagocytic capacity, ROS production, and bactericidal activity (34).

Diabetic mice presented hyperglucagonemia in addition to increased proportions of neutrophils expressing GcgR in the circulation. Furthermore, diabetic mice showed reduced neutrophil migration to the peritoneal cavity, accompanied by decreased bacterial clearance after the CLP procedure. In diabetic animals, sepsis reduced mice survival rate and raised glycemia. Intraperitoneal treatment with GcgR antagonist restored neutrophil accumulation and bacterial clearance in the peritoneal cavity, improving survival rate of diabetic and septic mice, without altering neither glycemia and blood neutrophil numbers, nor the levels of neutrophil relate chemokine (CXCL1/KC) in peritoneal cavity and circulation, or pro-inflammatory cytokine, IL-6, in peritoneum. Therefore, the restoration of neutrophil migration to the peritoneal cavity induced by GcgR antagonist treatment appears to be independent on the production of molecules that stimulate neutrophil activation. Together, our data suggest that glucagon reduces neutrophil migration to the injured tissue of diabetic mice by a direct effect on neutrophil migration machinery and not by the inhibition of neutrophil specific chemoattractants production. In addition, the GcgR antagonist improved diabetic mice survival rate without altering glycemia nor systemic inflammation induced by sepsis. These results strongly suggest that GcgR pathway might be over-activated, and thus, contributing to sepsis pathophysiology in diabetic mice.

As we observed that GcgR antagonism reestablished neutrophil migration in the peritoneal cavity of septic diabetic mice, we thought to evaluate the effect of glucagon on neutrophil migration *in vivo* and *in vitro*. In this work, we showed that glucagon inhibited CXCL1/KC-induced neutrophil migration to the peritoneal cavity of mice. In addition, we also noted that glucagon decreased the chemotaxis of murine neutrophils induced by CXCL1/KC, PAF, or fMLP *in vitro* as well as CXCL8/IL-8-induced chemotaxis of human circulating neutrophils *in vitro*. Since glucagon inhibited neutrophil chemotaxis toward several chemoattractant *in vitro*, it probably inhibited neutrophil migration through some molecular mechanisms independent of chemokine receptor down-regulation, as it has been previously reported for neutrophils obtained from diabetic mice (35). Importantly, neutrophils obtained from BM and blood of mice, as well as from human blood express GcgR. Interestingly, the percentage of neutrophils from blood that express GcgR is higher in humans than in mice. Beyond the possible differences that may exist between both species, the influence of circadian cycles in neutrophil heterogeneity may also be an explanation to this phenomenon. Since mice and humans have opposite circadian cycles and leukocyte isolations from both sources were performed during morning time, the samples may present differences related to the circadian regulation (36, 37).

To evaluate how glucagon inhibits the migration of mice neutrophil, we explored the molecular mechanisms that allow the recruitment of neutrophils to the site of infection. First, we assessed the surface translocation of two crucial integrins, CD11a and CD11b, involved with firm adhesion of neutrophils to endothelial cells, which culminate with the transmigration of these leukocytes from blood to the tissue (38–40). We noted that glucagon did not alter the CXCL1/KC- induced translocation of both CD11a and CD11b to the surface of mice’s neutrophil. The polymerization of actin filaments, the reorganization of the cytoskeletal network, and the polarization of the cells are major contributors that orchestrate the mechanical properties of neutrophils during the migration (41). We showed that glucagon inhibited CXCL1/KC-induced actin polymerization of neutrophils *in vitro*, indicating that glucagon reduces neutrophil migration through the impairment of polymerization of actin filaments. Besides, glucagon reduced chemotaxis and adhesion of human neutrophils to endothelial cells *in vitro*, without affecting CD11a and CD11b translocation, as it was observed in mice neutrophils. Thus, glucagon may affect the downstream signaling of integrins in neutrophils. As CXCL8/IL-8 production by human neutrophils was not affected by treatment with glucagon, chemokine production by human neutrophils may not be regulated by cAMP-PKA signaling pathway.

The glucagon receptor is a 7TM receptor that activates a Gs protein and consequently increases the intracellular levels of cAMP (12). In this work, we showed that GcgR antagonism, adenylyl cyclase inhibition, and PKA blockage restored the inhibitory effect of glucagon on CXCL1/KC-induced chemotaxis of neutrophils *in vitro*. Furthermore, glucagon directly stimulated PKA phosphorylation in neutrophils. Taken together, our data indicate that the impairment of neutrophil migration induced by glucagon *in vitro* depends on the activation of its receptor and the cAMP/PKA downstream signaling pathway. The effect of intracellular levels of cAMP and PKA activation on neutrophil chemotaxis is still a matter of controversy. Some pieces of evidence showed that the cAMP/PKA pathway is important to neutrophil migration (42, 43), while others indicate that signaling through this pathway impaired chemotaxis (44, 45). The literature suggests that depending on the intracellular cAMP levels, PKA activation on neutrophils may favor or inhibit actin polymerization and, consequently, neutrophil chemotaxis (46, 47). Our data indicate that PKA phosphorylation induced by glucagon in neutrophils inhibits actin polymerization, reducing neutrophil chemotaxis.

In addition to chemotaxis, other neutrophil functions are also altered in diabetes, such as phagocytosis, bactericidal activity, and ROS production, which contribute to the high incidence and severity of infections in diabetic patients (48). Thus, we explored whether glucagon could impact ROS generation by neutrophils *in vitro* and observed that it inhibited zymosan A-induced ROS production by murine neutrophils *in vitro*. This result suggest that glucagon may also be involved in the impaired bacterial clearance that diabetic and septic mice presented in the peritoneal cavity. In addition, this data contributes to explain the reduction in the CFU count in the peritoneum cavity promoted by treatment with GcgR antagonist on diabetic and septic animals, which culminated with an increased survival rate.

One limitation of our work is that alloxan-induced diabetes does not induce an autoimmune response in the β-pancreatic cells, as observed in type 1 diabetic patients (49). Nevertheless, diabetes induction in mice by alloxan exhibits several clinical conditions noted in patients. These include hyperglycemia, hypoinsulinemia, retinopathy, neuropathy, impaired wound healing, greater severity of sepsis, and mortality, when uncontrolled, among others (7, 50). Correspondingly, in our mice model of diabetes, the animals presented hyperglucagonemia. These diabetic mice also showed a failure of neutrophil migration, increased bacterial loads in the peritoneal cavity and reduced survival rate when submitted to CLP, which was also observed in NOD mice that spontaneously develop type 1 diabetes (8). In addition, although it has been reported that diabetic mice present higher levels of aspartate aminotransferase and alanine aminotransferase upon CLP-induction compared to non-diabetic mice (7), in our work we did not explore the effect of glucagon on liver injury or metabolism since it was not the main focus of our research. Finally, our evidence showing the involvement of cAMP-PKA pathway in the inhibitory effect of glucagon on neutrophil migration is also limited, since we only perform *in vitro* experiments. Therefore, we cannot claim that are a direct relationship between the activation of cAMP-PKA pathway induced by glucagon in neutrophils with the reduction of those recruitment *in vivo* in diabetic mice after CLP. On the other hand, the strength point of our work is that the expression of GcgR on neutrophils, and inhibition of migration in these cells by glucagon *in vitro*, was reproducible in human neutrophils. This gives our data a potential translation for the understanding of the mechanism responsible for the greater susceptibility to sepsis by diabetic patients.

The impairment of neutrophil migration is considered as a key factor in the severity of sepsis and the inefficient resolution of infections observed in diabetic patients (7, 51). In addition, the severity of sepsis has a close relationship with increased circulating levels of glucagon (10). In this context, our results could help to explain, at least in part, a possible mechanism associated with the higher severity of sepsis often seen in diabetic patients.

Altogether, our results demonstrate that GcgR antagonism restores neutrophil migration towards the site of infection, reestablishing local bacterial clearance in diabetic and septic mice. Also, glucagon-induced impairment of neutrophil migration *in vitro* is associated with an increase in the cAMP levels and PKA activation. Based on these findings, it would be interesting to study new therapeutic strategies for sepsis, targeting GcgR, to reestablish the migration and activity of neutrophils and thus control bacterial infection in septic and diabetic patients.

## Data Availability Statement

The original contributions presented in the study are included in the article/**Supplementary Material**. Further inquiries can be directed to the corresponding author.

## Ethics Statement

All human protocols were approved by the Research Ethics Committee of the Sergio Arouca National School of Public Health (ENSP, FIOCRUZ), protocol number 1.057.012. The patients/participants provided their written informed consent to participate in this study. The animal study was reviewed and approved by Committee on Use of Laboratory Animals of the Oswaldo Cruz Institute (CEUA-IOC/FIOCRUZ, licenses L-027/2016 and LW36/10).

## Author Contributions

DI and MF contributed to conception and design of the study, performed experiments, analyzed the data, and wrote the manuscript. CG-d-A contributed to design of the study, performed experiments, analyzed the data, and contributed to preparation of the manuscript. AC and AYOS performed experiments and analyzed the data. HC-F-N contributed to the conception and design of the study, with essential reagents or tools, analyzed the data, and contributed to preparation of the manuscript. RS contributed to design of the study, performed experiments, and analyzed the data. TF analyzed the data and contributed with essential reagents or tools. PRS and MM analyzed the data, contributed with essential reagents or tools and to preparation of the manuscript. ARS contributed to conception and design of the study, performed experiments, analyzed the data, contributed with essential reagents or tools and to preparation of the manuscript. VC contributed to conception and design of the study, with essential reagents or tools, analyzed the data, and wrote the manuscript. All authors contributed to the article and approved the submitted version.

## Funding

This study was financial supported by Fundação Carlos Chagas de Amparo à Pesquisa do Estado do Rio de Janeiro (FAPERJ), Conselho Nacional de Desenvolvimento Científico e Tecnológico (CNPq), Programa de Auxílio à Pesquisa (PAPESVI/FIOCRUZ), Programa INOVA FIOCRUZ, and Ministério da Saúde, Brazil and Coordenação de Aperfeiçoamento de Pessoal de Nível Superior - Brasil (CAPES).

## Conflict of Interest

The authors declare that the research was conducted in the absence of any commercial or financial relationships that could be construed as a potential conflict of interest.

## Abbreviations

AUC, area under the curve; BM, Bone marrow; cAMP, cyclic adenosine monophosphate; CFU, Colony-forming units; CLP, Cecum ligation and puncture; CREB, cAMP response element-binding; FBS, Fetal bovine serum; fMLP, N-formyl methionyl-leucyl-phenylalanine; GcgR, Glucagon receptor; HBSS, Hank’s balanced salt solution without Ca^+2^ or Mg^+2^; HUVEC, Human umbilical vein endothelial cell; LPS, Lipopolysaccharide; MFI, Median fluorescence intensity; P, Probability values; PAF, Platelet-activating factor; PDE, Phosphodiesterase; PKA, Protein kinase A; pPKA, PKA phosphorylation; SEM, Standard error of the mean; SM, Supplementary material.
