# Supplementary material for: Glucagon Reduces Neutrophil Migration and Increases Susceptibility to Sepsis in Diabetic Mice
Source: Front Immunol. 2021 Jul 6;12:633540. doi: 10.3389/fimmu.2021.633540 (PMC8290340; doi:10.3389/fimmu.2021.633540)
Supplement: Supplementary file 1 [file DataSheet_1.docx]

Supplementary Material (SM)

# Material and Methods

**Animals’ welfare**

We assessed mice’s welfare through observation of piloerection, respiratory rate, locomotion, feces alteration, water consumption, and amount of urine. Because diabetic animals showed an increase in water consumption and urine excretion rate, we provided new water and exchanged animal cages for clean ones every day. However, no alterations in piloerection, respiratory rate, locomotion, and feces alteration were noted in our animals.


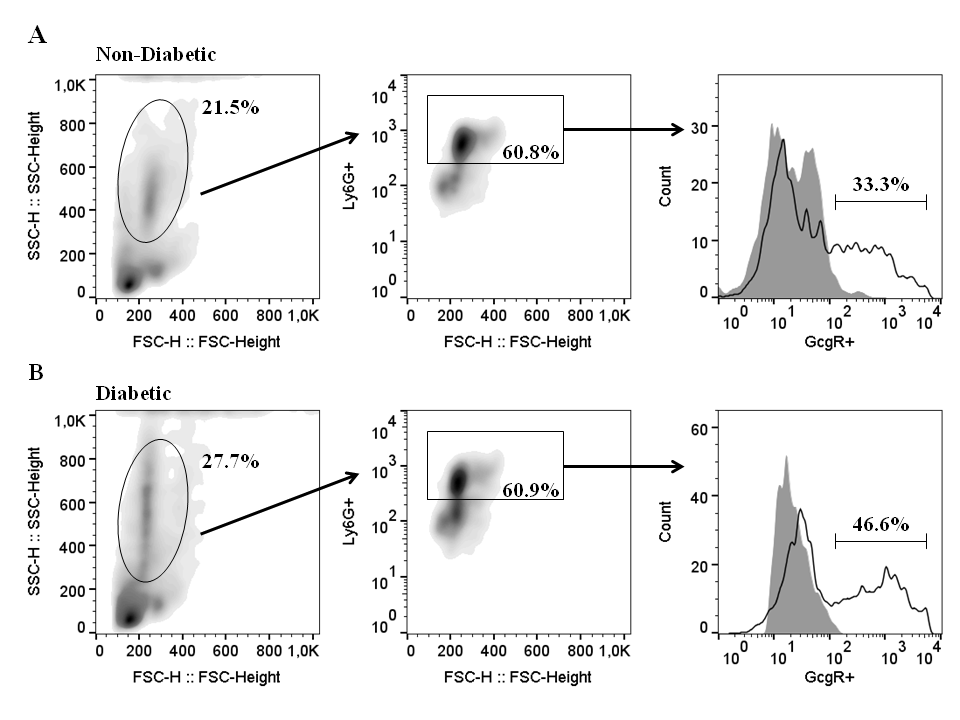
**Results**

**Figure S1. Gating strategies to identify the expression of GcgR on neutrophils obtained from the blood of non-diabetic and diabetic mice.** Density plots and histogram of cells populations from the blood of non-diabetic (A) and diabetic (B) animals. For the identification of neutrophils, cells from mice blood were stained with monoclonal antibody anti-Ly6G (clone 1A8) (FITC). To evaluate GcgR expression in neutrophils, we incubated cells with primary polyclonal rabbit anti-GcgR antibody and next with polyclonal anti-rabbit-Alexa 647 antibody. Analysis of GcgR expression were performed only on Ly6G^+^ cells. Isotype-matched antibodies were used to control nonspecific binding. Furthermore, to control nonspecific binding of GcgR, some samples were incubated only with anti-rabbit-Alexa 647 and showed less than 3% of positive events in all experiments and experimental groups. All antibodies were validated by the producers and experimenters. GcgR = Glucagon receptor.


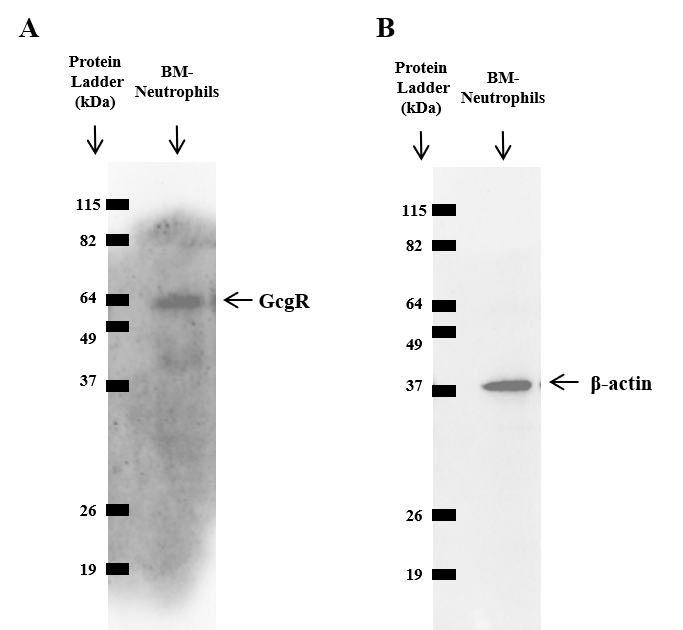


**Figure S2. Western blot evaluation of GcgR expression in murine Bone marrow (BM)-neutrophils.** Representative full-length blots of GcgR (A) and β-actin (B). Neutrophils were isolated from mice BM using a discontinuous gradient of percoll. Then, analysis of GcgR and β-actin expression were performed by western blot. All antibodies were validated by the producers and experimenters. GcgR = Glucagon receptor.

**
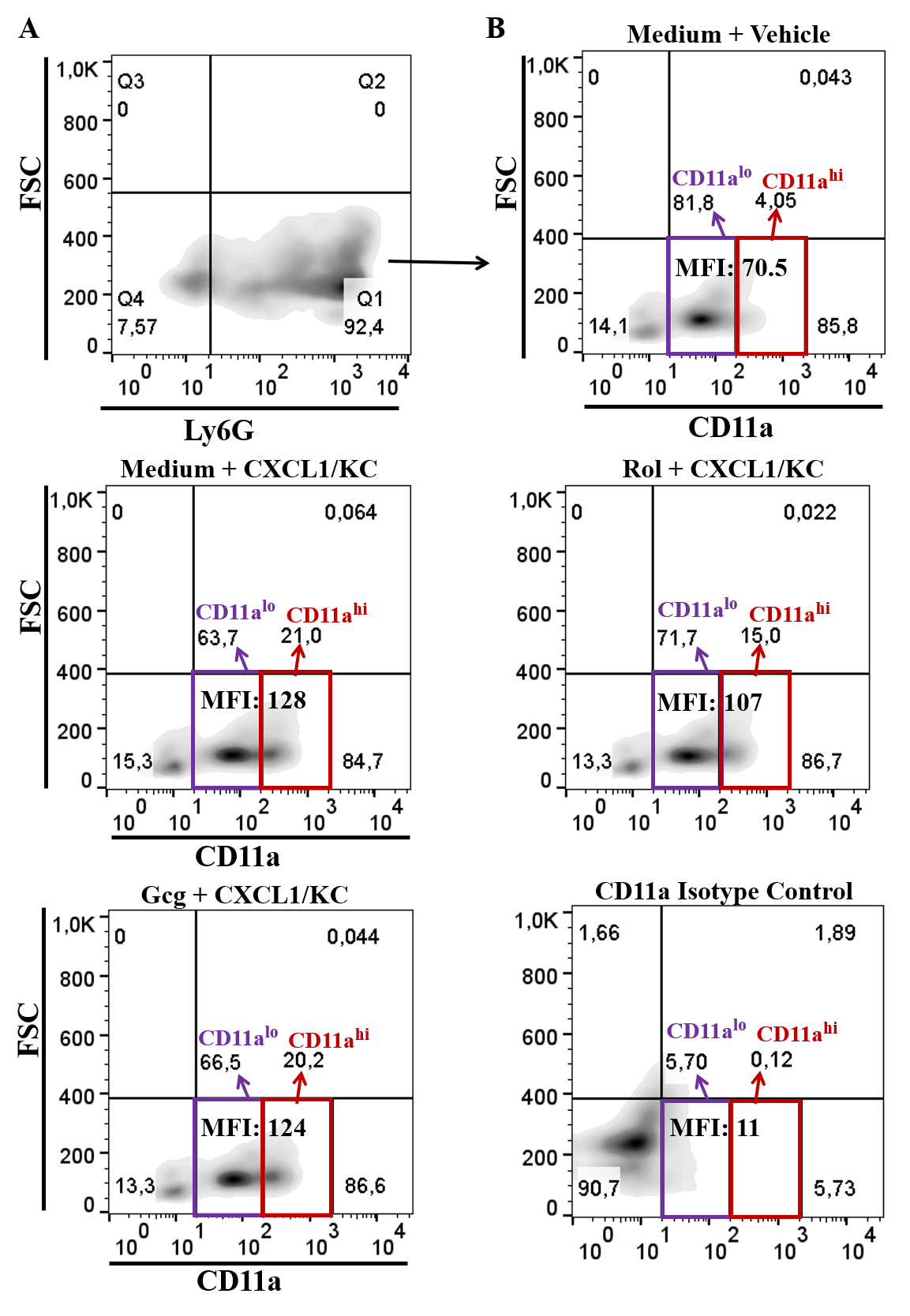
**

**Figure S3****. Gating strategies to evaluate the expression of CD11a on murine BM-neutrophils surface.** Representative density plots of Ly6G (A) and CD11a (B) expression on murine BM-neutrophils surface. Neutrophils were isolated from mice BM using a discontinuous gradient of percoll, and then, treated with medium, rolipram (5 µM) or glucagon (3 µM) for 30 min and stimulated with CXCL1/KC (10 nM) or vehicle for 40 min *in vitro*. Next, these cells were stained with monoclonal antibody anti-Ly6G (clone 1A8) (FITC) for identification of neutrophils. To evaluate CD11a expression on neutrophils surface, we incubated cells with monoclonal antibody anti-CD11a (PE) for 30 min. Analysis of CD11a expression were performed only on Ly6G^+^ cells. Isotype-matched antibody was used to control nonspecific binding. All antibodies were validated by the producers and experimenters. Gcg = Glucagon. Rol = Rolipram. MFI = Median fluorescence intensity. lo = low. hi= high.


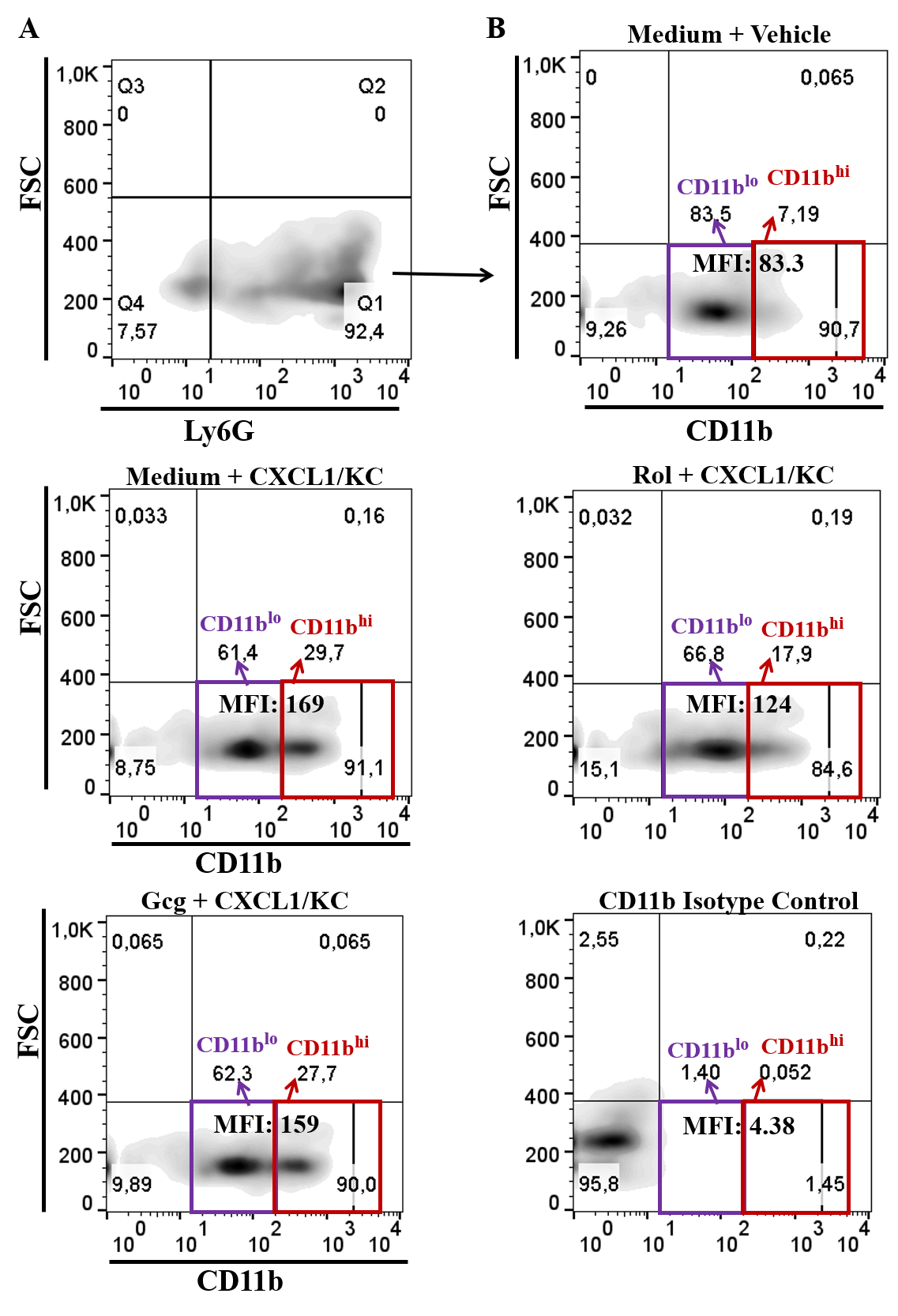


**Figure S4. Gating strategies to evaluate the expression of CD11b on murine BM-neutrophils surface.** Representative density plots of Ly6G (A) and CD11b (B) expression on murine BM-neutrophils surface. Neutrophils were isolated from mice BM using a discontinuous gradient of percoll, and, then, treated with medium, rolipram (5 µM) or glucagon (3 µM) for 30 min and stimulated with CXCL1/KC (10 nM) or vehicle for 40 min *in vitro*. Next, these cells were stained with monoclonal antibody anti-Ly6G (clone 1A8) (FITC) for identification of neutrophils. To evaluate CD11b expression on neutrophils surface, we incubated cells with monoclonal antibody anti-CD11b (PECY7) for 30 min. Analysis of CD11b expression were performed only on Ly6G^+^ cells. Isotype-matched antibody was used to control nonspecific binding. All antibodies were validated by the producers and experimenters. Gcg = Glucagon. Rol = Rolipram. MFI = Median fluorescence intensity. lo = low. hi= high.

**
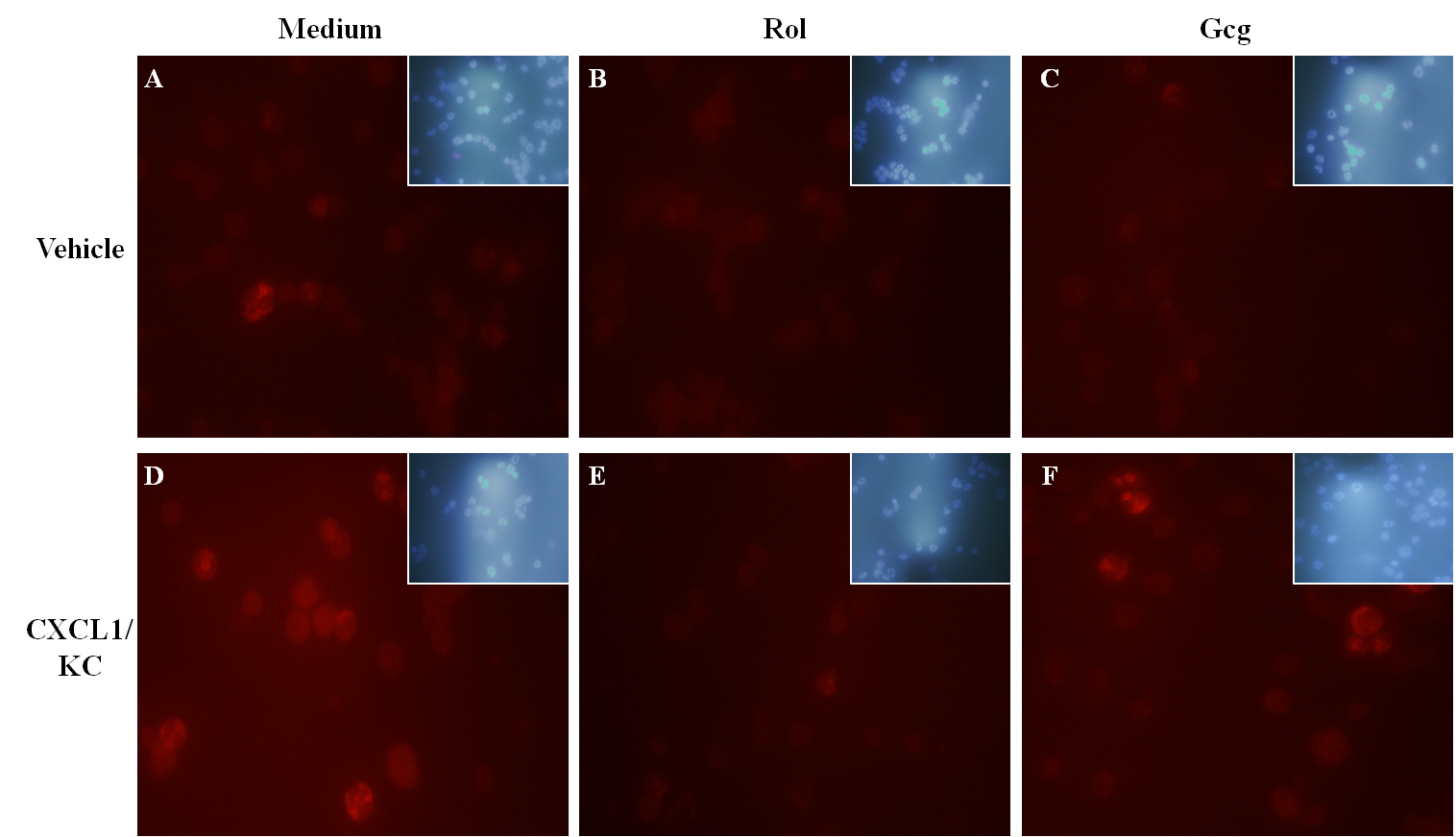
**

**Figure S5. Representative photomicrographs of F-actin content by cytofluorescence in murine BM-neutrophils.** Representative images of actin filaments stained with TRITC-phalloidin (red). In the inserts, we show the nucleus stained with DAPI (blue). Neutrophils were isolated from mice BM using a discontinuous gradient of percoll, and then treated with medium, rolipram (5 µM) or glucagon (3 µM) for 30 min and stimulated with CXCL1/KC (10 nM) or vehicle for 40 min *in vitro*. (A) Neutrophil treated with medium and non-stimulated *in vitro*. (B) Neutrophil treated with rolipram (5 µM) and non-stimulated *in vitro*. (C) Neutrophil treated with glucagon (3 µM) and non-stimulated *in vitro*. (D) Neutrophil treated with medium and stimulated with CXCL1/KC (10 nM) *in vitro*. (E) Neutrophil treated with rolipram (5 µM) and stimulated with CXCL1/KC (10 nM) *in vitro*. (F) Neutrophil treated with glucagon (3 µM) and stimulated with CXCL1/KC (10 nM) *in vitro.*

**
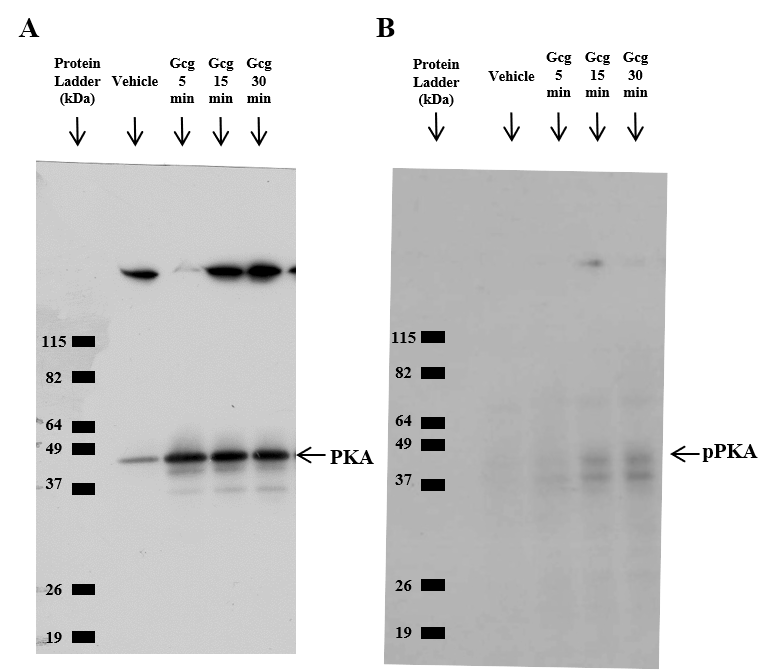
**

**Figure S6. Western blot evaluation of PKA and pPKA expression in murine BM-neutrophils.** Representative full-length blots of PKA (A) and pPKA (B). Neutrophils were isolated from mice BM using a discontinuous gradient of percoll, and then treated with vehicle or glucagon (3 µM) for 5, 15, or 30 min *in vitro*. Then, analysis of the PKA and pPKA expression were performed by western blot. All antibodies were validated by the producers and experimenters. Gcg = Glucagon. PKA = Protein kinase A. pPKA = PKA phosphorylation.


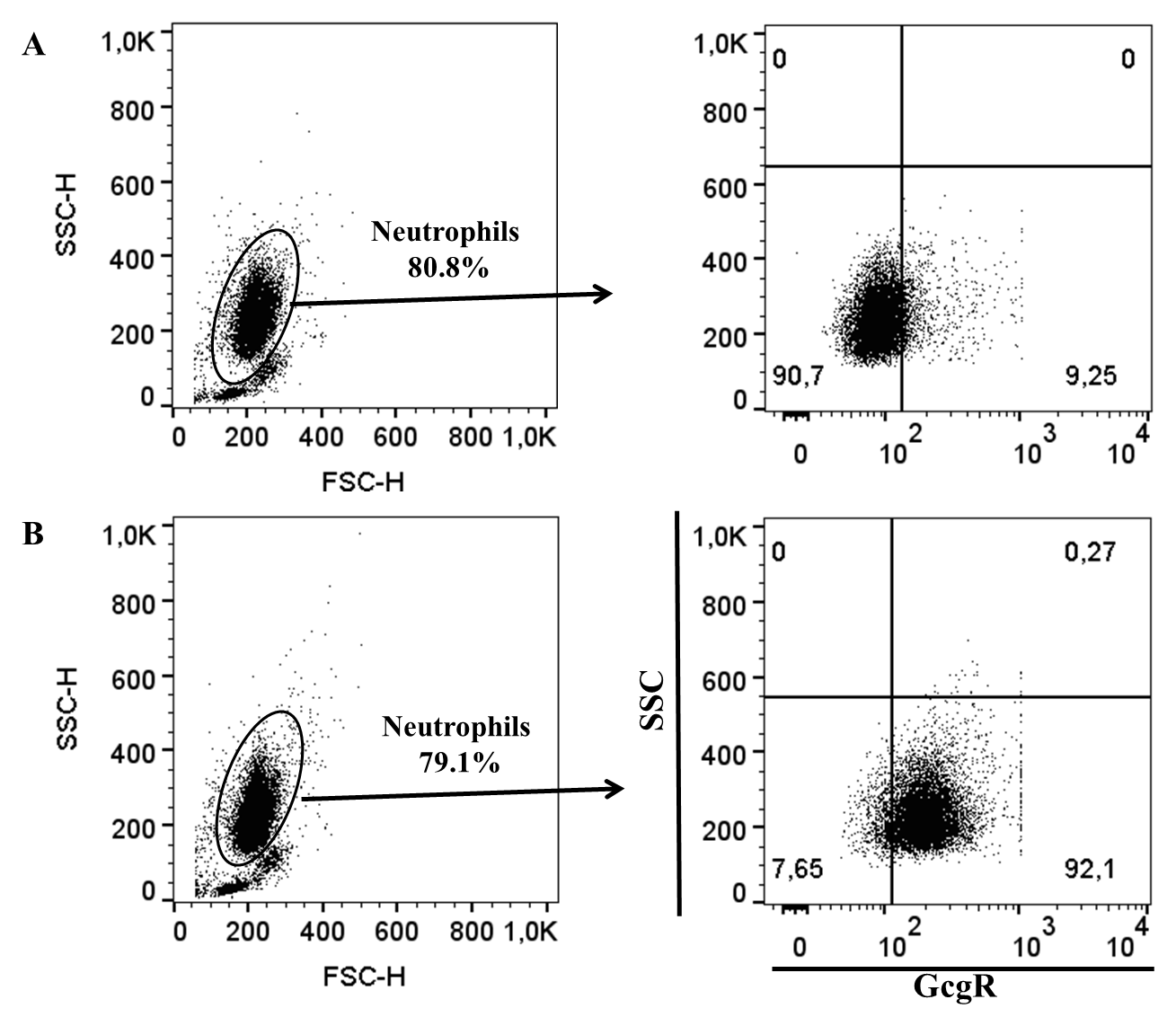


**Figure S7. Gating strategies to identify the expression of GcgR on neutrophils obtained from the human peripheral blood.** Representative density plots of control staining using isotype control antibody (A), and specific anti-GcgR (PE) antibody staining (B), in blood neutrophils. Neutrophils were isolated from human blood using Ficollpaque and, then, we performed the gated based on their FSC/SSC profile. To evaluate GcgR expression in isolated neutrophils, cells were incubated with polyclonal antibody anti-GcgR (PE). The isotype-matched antibody was used to control nonspecific binding and showed less than 10% of positive events in all samples. All antibodies were validated by the producers and experimenters. GcgR = Glucagon receptor.

**
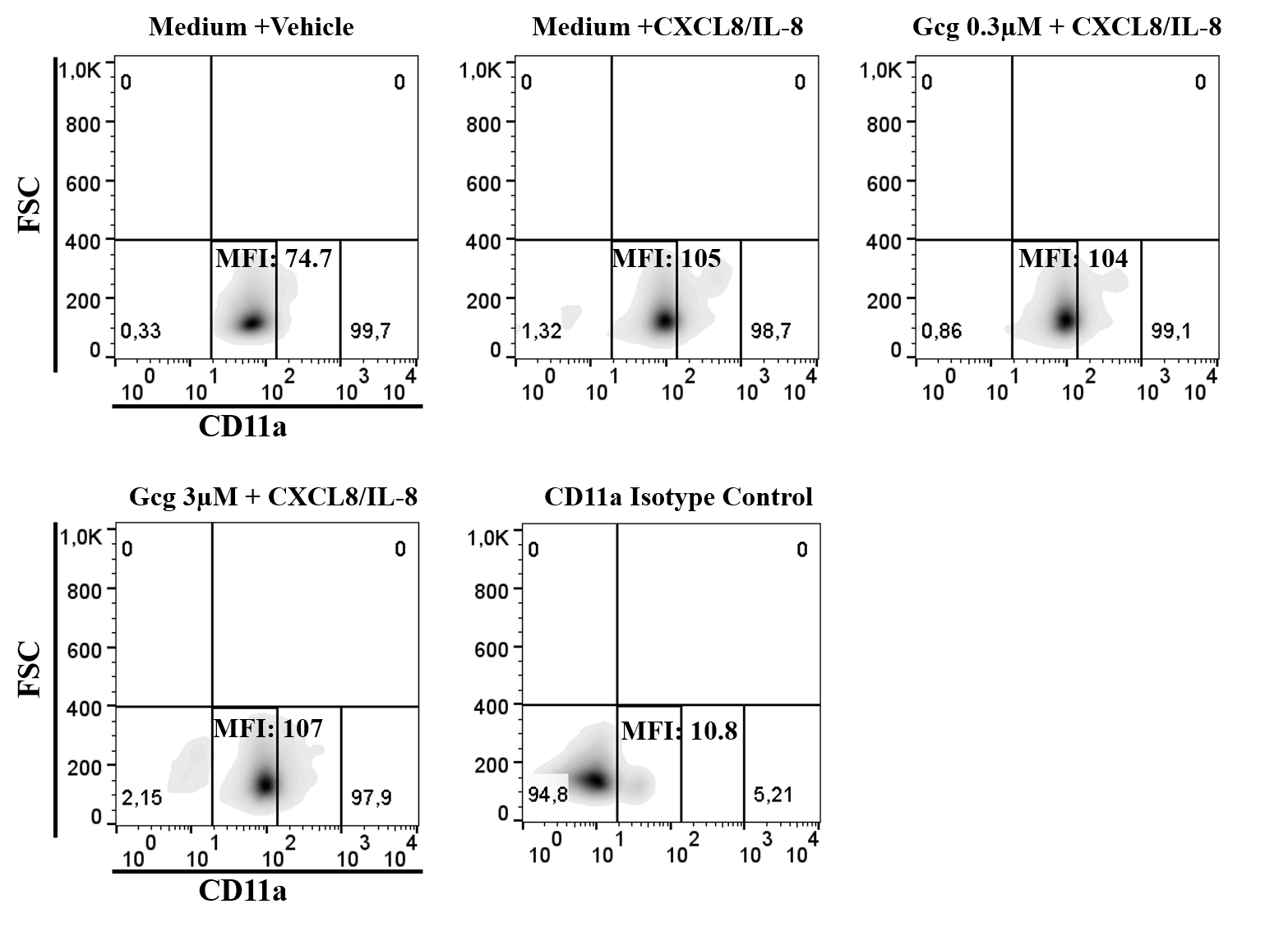
**

**Figure S8. Gating strategies to evaluate the expression of CD11a on surface of human neutrophils obtained from blood.** Representative density plots of CD11a expression on human neutrophils surface. For identification of neutrophils, cells were isolated from human blood using Ficollpaque, and then, treated with medium or glucagon (0.3 or 3 µM) for 40 min and stimulated with CXCL8/IL-8 (12 nM) or vehicle for 1h *in vitro*. Next, these cells were stained with monoclonal antibody anti-Ly6G (clone 1A8) (FITC) for identification of neutrophils. To evaluate CD11a expression on neutrophils surface, we incubated cells with monoclonal antibody anti-CD11a (PE) for 30 min. Analysis of CD11a expression were performed only on Ly6G^+^ cells. Isotype-matched antibodies were used to control nonspecific binding. All antibodies were validated by the producers and experimenters. Gcg = Glucagon. MFI = Median of intensity fluorescent.


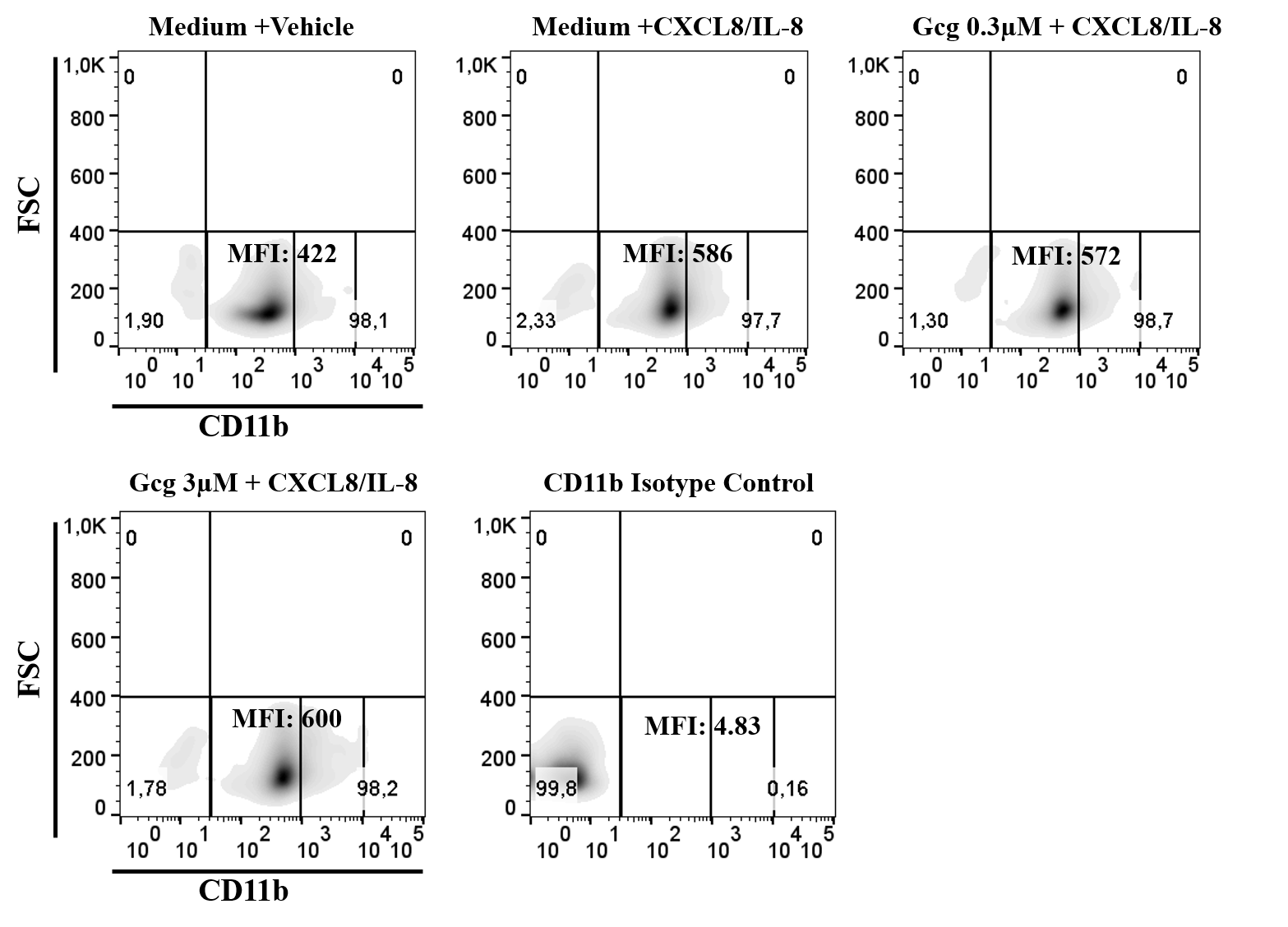


**Figure S9. Gating strategies to evaluate the expression of CD11b on surface of human neutrophils obtained from blood.** Representative density plots of CD11b expression on human neutrophils surface. For identification of neutrophils, cells were isolated from human blood using Ficollpaque, and then, treated with medium or glucagon (0.3 or 3 µM) for 40 min and stimulated with CXCL8/IL-8 (12 nM) or vehicle for 1h *in vitro*. Next, these cells were stained with monoclonal antibody anti-Ly6G (clone 1A8) (FITC) for identification of neutrophils. To evaluate CD11b expression on neutrophils surface, we incubated cells with monoclonal antibodies anti-CD11b (PECY7) for 30 min. Analysis of CD11b expression were performed only on Ly6G^+^ cells. Isotype-matched antibodies were used to control nonspecific binding. All antibodies were validated by the producers and experimenters. Gcg = Glucagon. MFI = Median of intensity fluorescent.
